# Discharge protocol in acute pancreatitis: an international survey and cohort analysis

**DOI:** 10.1038/s41598-023-48480-z

**Published:** 2023-12-13

**Authors:** Rita Nagy, Klementina Ocskay, Zoltán Sipos, Andrea Szentesi, Áron Vincze, László Czakó, Ferenc Izbéki, Natalia V. Shirinskaya, Vladimir L. Poluektov, Alexandr N. Zolotov, Yin Zhu, Liang Xia, Wenhua He, Robert Sutton, Peter Szatmary, Rajarshi Mukherjee, Isobel Saffron Burridge, Emma Wauchope, Elsa Francisco, David Aparicio, Bruno Pinto, António Gomes, Vitor Nunes, Vasile Marcel Tantau, Emanuela Denisa Sagau, Alina Ioana Tantau, Andra Iulia Suceveanu, Cristina Tocia, Andrei Dumitru, Elizabeth Pando, Piero Alberti, Arturo Cirera, Xavier Molero, Hong Sik Lee, Min Kyu Jung, Eui Joo Kim, Sanghyub Lee, María Lourdes Ruiz Rebollo, Reyes Busta Nistal, Sandra Izquierdo Santervas, Dusan Lesko, Marek Soltes, Jozef Radonak, Hubert Zatorski, Ewa Małecka-Panas, Adam Fabisiak, M. Susak Yaroslav, V. Maksymenko Mykhailo, A. Tkachenko Olekcandr, Giedrius Barauskas, Vytautas Simanaitis, Povilas Ignatavicius, Mariana Jinga, Vasile-Daniel Balaban, Cristina Patoni, Liang Gong, Kai Song, Yunlong Li, T. Cúrdia Gonçalves, Marta Freitas, Vítor Macedo, Marlies Vornhuelz, Sarah Klauss, Georg Beyer, Aydin Seref Koksal, Mukaddes Tozlu, Ahmet Tarik Eminler, Nuria Torres Monclús, Eva Pijoan Comas, Juan Armando Rodriguez Oballe, Łukasz Nawacki, Stanisław Głuszek, Alberto Rama-Fernández, Marco Galego, Daniel de la Iglesia, Umut Emre Aykut, Deniz Güney Duman, Rahmi Aslan, Adriana Gherbon, Lihui Deng, Wei Huang, Qing Xia, Goran Poropat, Anja Radovan, Luka Vranić, Claudio Ricci, Carlo Ingaldi, Riccardo Casadei, Ionut Negoi, Cezar Ciubotaru, Florin Mihail Iordache, Gabriel Constantinescu, Vasile Sandru, Engin Altintas, Hatice Rizaoglu Balci, Júlio Constantino, Débora Aveiro, Jorge Pereira, Suleyman Gunay, Seda Misirlioglu Sucan, Oleksiy Dronov, Inna Kovalska, Nikhil Bush, Surinder Singh Rana, Serge Chooklin, Serhii Chuklin, Ionut Adrian Saizu, Cristian Gheorghe, Philipp Göltl, Michael Hirth, Radu Bogdan Mateescu, Geanina Papuc, Georgi Angelov Minkov, Emil Tihomirov Enchev, Laura Mastrangelo, Elio Jovine, Weiwei Chen, Quping Zhu, Anita Gąsiorowska, Natalia Fabisiak, Mihailo Bezmarevic, Andrey Litvin, Martina Cattani Mottes, Eun Kwang Choi, Peter Bánovčin, Lenka Nosáková, Mila Dimitrova Kovacheva-Slavova, Ali Kchaou, Ahmed Tlili, Marco V. Marino, Katarzyna Kusnierz, Artautas Mickevicius, Marcus Hollenbach, Pavol Molcan, Orestis Ioannidis, Mark Valerievich Tokarev, Ali Tüzün Ince, Ivan Albertovich Semenenko, Shamil Galeev, Elena Ramírez-Maldonado, Ville Sallinen, Petr Pencik, Judit Bajor, Patricia Sarlós, Roland Hágendorn, Szilárd Gódi, Imre Szabó, József Czimmer, Gabriella Pár, Anita Illés, Nándor Faluhelyi, Péter Kanizsai, Tamás Nagy, Alexandra Mikó, Balázs Németh, József Hamvas, Barnabás Bod, Márta Varga, Imola Török, János Novák, Árpád Patai, János Sümegi, Csaba Góg, Mária Papp, Bálint Erőss, Szilárd Váncsa, Brigitta Teutsch, Katalin Márta, Péter Jenő Hegyi, Tamás Tornai, Balázs Lázár, Tamás Hussein, Dorottya Tarján, Mónika Lipp, Beáta Kovács, Orsolya Urbán, Emese Fürst, Edina Tari, Ibolya Kocsis, Pál Maurovich-Horvát, Balázs Tihanyi, Orsolya Eperjesi, Zita Kormos, Pál Ákos Deák, Andrea Párniczky, Péter Hegyi

**Affiliations:** 1 Centre for Translational Medicine, Semmelweis University, Budapest, Hungary; 2 Institute for Translational Medicine, Medical School, University of Pécs, Pécs, Hungary; 3 Heim Pál National Pediatric Institute, Budapest, Hungary; 4 Division of Gastroenterology, First Department of Medicine, Medical School, University of Pécs, Pécs, Hungary; 5 Department of Medicine, University of Szeged, Szeged, Hungary; 6 Szent György University Teaching Hospital of Fejér County, Székesfehérvár, Hungary; 7 Omsk State Medical Information-Analytical Centre, Omsk State Medical University, Omsk, Russia; 8 Department of Surgery and Urology, Omsk State Medical University, Omsk, Russia; 9 Department of Pathophysiology, Clinical Pathophysiology, Omsk State Medical University, Omsk, Russia; 10 Department of Gastroenterology, First Affiliated Hospital of Nanchang University, Nanchang, China; 11 University of Liverpool, Liverpool University Hospitals NHS Foundation Trust, Liverpool, UK; 12 Liverpool University Hospitals NHS Foundation Trust, Liverpool, UK; 13 Surgery Department, Hospital Prof. Ferndo Fonseca, Amadora, Portugal; 14 “Octavin Fodor” Institute of Gastroenterology and Hepartology, “Iuliu Hatieganu” University of Medicine and Pharmacy, Cluj Napoca, Romania; 15 Gastroenterology Department, 4th Medical Clinic, “Iuliu Hatieganu” University of Medicine and Pharmacy, Cluj Napoca, Romania; 16 Faculty of Medicine, Ovidius University of Constanta, Constanta, Romania; 17 Department of Hepato-Pancreato-Biliary and Transplant Surgery, Hospital Universitari Vall d’Hebron, Universitat Autònoma de Barcelona, Barcelona, Spain; 18 Exocrine Pancreas Research Unit, Hospital Universitari Vall d’Hebron, Institut de Recerca, Universitat Autònoma de Barcelona, CIBEREHD, Barcelona, Spain; 19 Division of Gastroenterology and Hepatology, Department of Internal Medicine, Korea University Anam Hospital, Seoul, Republic of Korea; 20 Division of Gastroenterology, Department of Internal Medicine, Gachon University Gil Medical Center, Gachon University College of Medicine, Incheon, Republic of Korea; 21 Department of Internal Medicine and Liver Research Institute, Seoul National University Hospital, Seoul, Republic of Korea; 22 Servicio de Aparato Digestivo Hospital Clínico Universitario Valladolid, Valladolid, Spain; 23 1st Department of Surgery, University Hospital of L. Pasteur, Kosice, Slovak Republic; 24 Department of Digestive Tract Diseases, Medical University of Lodz, Lodz, Poland; 25 Department of Surgery With a Course of Emergency and Vascular Surgery, Bogomolet National Medical University, Kiev, Ukraine; 26 Kyiv City Clinical Emergency Hospital, Kiev, Ukraine; 27 Department of Surgery, Lithuanian University of Health Sciences, Kaunas, Lithuania; 28 “Carol Davila” University of Medicine and Pharmacy, Bucharest, Romania; 29 Department of Gastroenterology, Peking Union Medical College Hospital, Chinese Academy of Medical Sciences & Peking Union Medical College, Beijing, China; 30 Gastroenterology Department, Hospital da Senhora da Oliveira, Guimarães, Portugal; 31 Life and Health Sciences Research Institute (ICVS), School of Medicine, University of Minho, Braga/Guimarães, Portugal; 32 ICVS/3B’s–PT Government Associate Laboratory, Braga/Guimarães, Portugal; 33 LMU University Hospital, LMU Munich, Munich, Germany; 34 Department of Gastroenterology, Faculty of Medicine, Sakarya University, Sakarya, Turkey; 35 University Hospital Arnau de Vilanova, Hospital University Santa Maria, Lleida, Spain; 36 Collegium Medicum, The Jan Kochanowski University in Kielce, Kielce, Poland; 37 Gastroenterology Department, University Hospital of Santiago de Compostela, Santiago de Compostela, Spain; 38 Marmara University Education and Training Hospital, Istanbul, Turkey; 39 Discipline of Internal Medicine: Diabetes, Nutrition, Metabolic Diseases and Systemic Rheumatology, Victor Babeş University of Medicine and Pharmacy Timisoara, Timisoara, Romania; 40 Department of Integrated Traditional Chinese and Western Medicine, Sichuan Provincial Pancreatitis Center and West China-Liverpool Biomedical Research Center, West China Hospital, Sichuan University, Chengdu, China; 41 Department of Gastroenterology, Clinical Hospital Center Rijeka, University of Rijeka, Rijeka, Croatia; 42 Division of Pancreatic Surgery, IRCCS, Azienda Ospedaliero Universitaria di Bologna, Bologna, Italy; 43 Department of Internal Medicine and Surgery (DIMEC), Alma Mater Studiorum, University of Bologna, S. Orsola-Malpighi Hospital, Bologna, Italy; 44 Emergency Hospital of Bucharest, Carol Davila University of Medicine and Pharmacy Bucharest, Bucharest, Romania; 45 Gastroenterology Department, Faculty of Medicine, Mersin University, Yenisehir/Mersin, Turkey; 46 Unidade HBP, Serviço de Cirurgia Geral, Centro Hospitalar Tondela-Viseu, Viseu, Portugal; 47 İzmir Katip Çelebi University Atatürk Training and Research Hospital, Karabaglar/Izmir, Turkey; 48 General Surgery #1, Bogomolets National Medical University, Kiev, Ukraine; 49 Department of Gastroenterology, Postgraduate Institute of Medical Education and Research (PGIMER), Chandigarh, India; 50 Lviv Regional Clinical Hospital, Lviv, Ukraine; 51 Clinical Institute Fundeni, Bucharest, Romania; 52 Department of Medicine II, University Medical Center Mannheim, Medical Faculty Mannheim, Heidelberg University, Mannheim, Germany; 53 Gastroenterology Department, Colentina Clinical Hospital Bucharest, Bucharest, Romania; 54 Department of Surgery, University Hospital, Stara Zagora, Bulgaria; 55 Department of Surgery, AOU Sant’Orsola Malpighi, IRCCS Azienda Ospedaliera Universitaria, Bologna, Italy; 56 Department of Gastroenterology, Clinical Medical College, Yangzhou University, Yangzhou, Jiangsu China; 57 Department of Gastroenterology Medical, University of Lodz, Lodz, Poland; 58 Department for Hepatobiliary and Pancreatic Surgery, Clinic for General Surgery, Military Medical Academy, University of Defense, Belgrade, Serbia; 59 Gomel State Medical University, Gomel, Belarus; 60 Department of Medicine, Gastroenterology, The Pancreas Institute, G.B. Rossi University Hospital, Verona, Italy; 61 Department of Internal Medicine, Jeju National University College of Medicine, Jeju, South Korea; 62 Clinic of Internal Medicine - Gastroenterology, JFM CU, Jessenius Faculty of Medicine in Martin (JFM CU), Comenius University in Bratislava, Bratislava, Slovakia; 63 Department of Gastroenterology, Queen Yoanna University Hospital, Medical University of Sofia, Sofia, Bulgaria; 64 Habib Bourguiba University Hospital, Sfax, Tunisia; 65 Mohamed Ben Sassi Hospital, Gabes, Tunisia; 66 General Surgery Department, Azienda Ospedaliera Ospedali Riuniti Villa Sofia-Cervello, Palermo, Italy; 67 Vilnius University Hospital Santariskiu Klinikos, Vilnius, Lithuania; 68 Division of Gastroenterology, University of Leipzig Medical Center, Leipzig, Germany; 69 Hepatology and Gastroenterology Department of Roosevelt Hospital, Banska Bystrica, Slovakia; 70 4th Department of Surgery, Medical School, Aristotle University of Thessaloniki, General Hospital “George Papanikolaou”, Thessaloniki, Greece; 71 Sklifosovsky Institute for Clinical Medicine, Sechenov First Moscow State Medical University, Moscow, Russia; 72 Hospital of Bezmialem Vakif University, School of Medicine, Istanbul, Turkey; 73 Sechenov University, Moscow, Russia; 74 Saint Luke Clinical Hospital, St. Petersburg, Russia; 75 General Surgery, Consorci Sanitari del Garraf, Sant Pere de Ribes, Barcelona, Spain; 76 Department of Transplantation and Liver Surgery, Helsinki University Hospital and University of Helsinki, Helsinki, Finland; 77 Centrum péče o zažívací trakt, Vítkovická Nemocnice a.s., Ostrava, Czech Republic; 78 Department of Medical Imaging, Medical School, University of Pécs, Pécs, Hungary; 79 Department of Emergency Medicine, Medical School, University of Pécs, Pécs, Hungary; 80 Department of Laboratory Medicine, Medical School, University of Pécs, Pécs, Hungary; 81 Peterfy Hospital, Budapest, Hungary; 82 Dr. Bugyi István Hospital, Szentes, Hungary; 83 Department of Gastroenterology, BMKK Dr Rethy Pal Hospital, Békéscsaba, Hungary; 84 County Emergency Clinical Hospital of Târgu Mures - Gastroenterology Clinic and University of Medicine, Pharmacy, Sciences and Technology “George Emil Palade”, Targu Mures, Romania; 85 Pándy Kálmán Hospital of Békés County, Gyula, Hungary; 86 Markusovszky University Teaching Hospital, Szombathely, Hungary; 87 Borsod-Abaúj-Zemplén County Hospital and University Teaching Hospital, Miskolc, Hungary; 88 Healthcare Center of County Csongrád, Makó, Hungary; 89 Department of Gastroenterology, Institute of Internal Medicine, Faculty of Medicine, University of Debrecen, Debrecen, Hungary; 90 Institute of Pancreatic Diseases, Semmelweis University, Budapest, Hungary; 91 Department of Laboratory Medicine, Semmelweis University, Budapest, Hungary; 92 MTA-SE Cardiovascular Imaging Research Group, Medical Imaging Centre, Semmelweis University, Budapest, Hungary; 93 Department for Surgery, Hungarian Defence Forces - Medical Centre, Budapest, Hungary; 94 Medical Imaging Centre, Department of Radiology, Semmelweis University, Budapest, Hungary; 95 Translational Pancreatology Research Group, Interdisciplinary Centre of Excellence for Research Development and Innovation, University of Szeged, Szeged, Hungary

**Keywords:** Gastroenterology, Medical research

## Abstract

There are several overlapping clinical practice guidelines in acute pancreatitis (AP), however, none of them contains suggestions on patient discharge. The Hungarian Pancreatic Study Group (HPSG) has recently developed a laboratory data and symptom-based discharge protocol which needs to be validated. (1) A survey was conducted involving all members of the International Association of Pancreatology (IAP) to understand the characteristics of international discharge protocols. (2) We investigated the safety and effectiveness of the HPSG-discharge protocol. According to our international survey, 87.5% (49/56) of the centres had no discharge protocol. Patients discharged based on protocols have a significantly shorter median length of hospitalization (LOH) (7 (5;10) days vs. 8 (5;12) days) *p* < 0.001), and a lower rate of readmission due to recurrent AP episodes (*p* = 0.005). There was no difference in median discharge CRP level among the international cohorts (*p* = 0.586). HPSG-protocol resulted in the shortest LOH (6 (5;9) days) and highest median CRP (35.40 (13.78; 68.40) mg/l). Safety was confirmed by the low rate of readmittance (n = 35; 5%). Discharge protocol is necessary in AP. The discharge protocol used in this study is the first clinically proven protocol. Developing and testifying further protocols are needed to better standardize patients’ care.

## Introduction

The incidence of acute pancreatitis (AP) is continuously increasing worldwide with an approximate annual incidence of 13–45 new cases per 100,000, meaning a 30% rise in the past 2 decades^1,2^. The disease itself, especially the severe form may lead to a prolonged hospital stay which can be associated with adverse patient outcomes and high hospital occupancy^3^. Moreover, longer hospital stay can result in higher costs^4^. The estimated annual total cost for AP admissions reached $2.2 billion, with a mean cost per hospitalization of $9870 based on a nationwide analysis in the United States^5^.

In order to achieve the best treatment for a disease, it is obvious that evidence-based guidelines need to be used^6,7^. The currently used evidence-based medicine guidelines in AP focuses on the diagnosis and management of AP, without clear recommendation on patient discharge^8^. Consequently, discharge decisions are made based on local experts’ onsite opinions leading to a variety of discharge approaches. A few years ago, the Hungarian Pancreatic Study Group (HPSG) developed a discharge protocol, but it has not been extensively tested and compared with other local protocols.

In this study, our aim was to conduct a widespread international survey and investigate the safety (readmission rate) and effectiveness (length of hospital stay) of the HPSG-protocol.

## Methods and materials

### International cohort

To assess the worldwide trends in patient discharge in AP we conducted a multicentre web-based survey by following the Checklist for Reporting of Survey Studies (CROSS)^9^. We sent a letter of invitation and a questionnaire to all members of the International Association of Pancreatology (IAP) in January 2021. The questionnaire’s main purpose was (i) to investigate the presence of any discharge protocol in AP and (ii) to understand the laboratory parameters and the clinical status of the patients upon discharge. There was no pre-testing period for the questionnaire. In case the collaborators confirmed their participation in the project, we sent a second email with further details and a pre-defined Excel sheet to collect data on gender, age, aetiology, length of hospitalization (LOH), mortality and severity of AP, discharge C-reactive protein (CRP) and 1-month readmission rate. Overall, the international data were collected retrospectively. The participants were asked to upload the completed Excel sheet to a private Google Drive folder. The participants did not have access to other collaborators’ datasets. The timeframe of the survey took two months. To avoid multiple participation, we carefully checked the participating centres, departments, and affiliations. In case of any questions, the first author was in charge of keeping in contact. The detailed questionnaire and pre-defined Excel sheet can be found in the supplementary documents (Fig. S1, Table S1). For the statistical analysis, we divided the international centres based on the presence of discharge protocol, creating an international protocol and an international non-protocol cohort, and compared the relevant clinical outcomes, such as LOH, discharge CRP value, and readmission rate.

### The HPSG discharge protocol

In 2016, the HPSG developed a discharge protocol with specific and combined elements on clinical status, laboratory parameters, and therapy. The protocol was developed based on the C20 point of the IAP/APA and HPSG EBM guideline which indicated that oral feeding in predicted mild pancreatitis can be restarted as early as the intensity of abdominal pain and inflammatory markers have started to decline^8^. The protocol was as follows:

1. Patient’s CRP level and either amylase or lipase levels were monitored every day.
  - Once the patient’s abdominal pain resolved and
  - Pancreatic enzyme levels showed a decreasing trend and
  - CRP level started to decrease and
  - there was no clinical condition that contraindicated oral feeding, the patient’s oral feeding with solid diet was immediately started.
2. If, 24 h after oral refeeding,
  - the patient has not developed any abdominal symptoms and
  - the pancreatic enzyme level has decreased further and
  - there were no other conditions or therapies (e.g., iv. antibiotics, endoscopic intervention) requiring hospitalisation and
  - CRP level has
    (i) fallen below 50 mg/l, the patient was discharged
    (ii) further decreased but remained above 50 mg/l, both hospitalization and oral feeding were continued for an additional day
3. If, after the additional 24 h of oral feeding (i.e., 48 h after refeeding was started)
  - the patient has not developed any abdominal symptoms and
  - the pancreatic enzyme level has decreased further and
  - there was no clinical condition that contraindicated feeding and,
  - CRP level has further decreased, the patient was discharged independently of the absolute CRP value.

The CRP value of 50 mg/l has been arbitrarily set based on previous clinical experience and related literature^10–12^. As the role of CRP at discharge in acute pancreatitis has not been previously investigated, this is the first time we tested its role and the safety of this cut off value.

Three of the 17 investigated centres used the above-mentioned discharge protocol in Hungary. Therefore, for data analysis, two groups of the Hungarian cohort were identified: (1) centres where the HPSG-discharge protocol was used (688 patients – Hungarian protocol cohort) and (2) where no discharge protocol was used (941 patients – Hungarian non-protocol cohort). A multicenter, multinational, prospective AP registry developed in 2013 by HPSG was used for data analysis and patients were enrolled during the period 2016–2019. Diagnosis of acute pancreatitis and its severity was defined based on the Atlanta “two of three” classification: abdominal pain, pancreatic enzyme elevation at least three times above the upper limit and morphological changes^13^.

### Statistical analysis

Statistical analyses were performed by using R environment (R Core Team (2021), version 4.1.0). For descriptive statistics, the number of patients, mean, standard deviation (SD), minimum, median and maximum values were calculated for continuous variables and case number and percentage were calculated for categorical values. To determine statistical significance between two groups of independent samples, t-test was used for normally distributed data and the Mann–Whitney U and Mood’s test for non-normally distributed data. The association between categorical variables was calculated by the Chi-square test and Fisher’s exact test. “Pairwise Nominal Independence” post-hoc test (package: rcompanion) was conducted using Bonferroni correction for a 2-dimensional matrix of two categorical variables in which at least one dimension has more than two levels. Receiver operating characteristics (ROC) analysis was performed to assess the accuracy of the prediction of discharge CRP value in terms of 1-month readmission. The threshold of significance was *p* < 0.05.

### Ethics

The study was approved by the Scientific and Research Ethics Committee of the Medical Research Council (22254e1/2012/EKU, 17787-8/2020/EÜIG). The study was performed in accordance with the Declaration of Helsinki and all patients provided written informed consent. Patients’ data of foreign centres were treated entirely anonymously.

## Results

### Basic characteristics of the international cohorts

**Figure 1:**
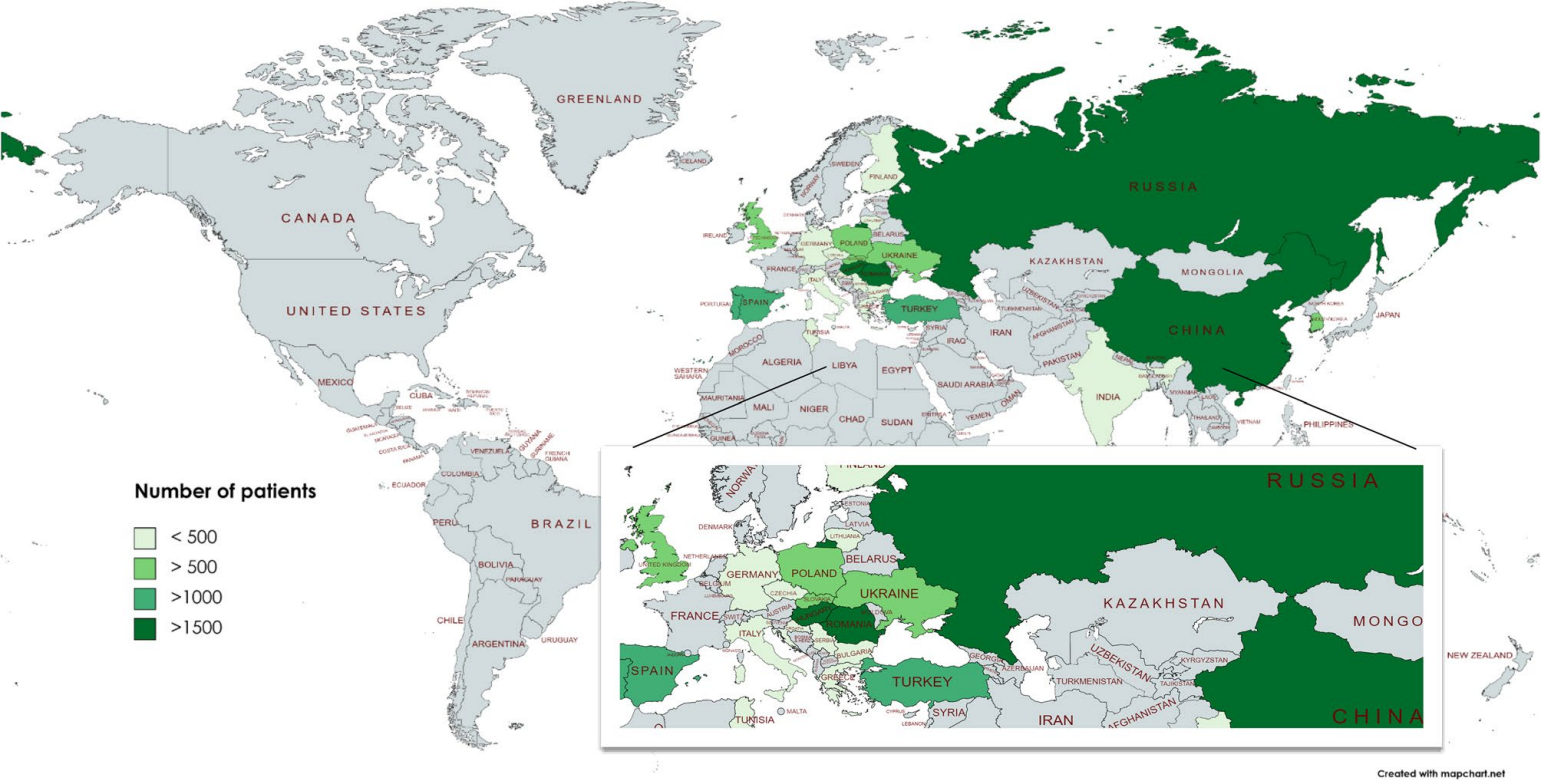
Map of the participating countries in the survey and analysis. The map shows the number of patients provided for analysis in different shades of green. A darker shade indicates more patients included. Created by MapChart (https://mapchart.net/world.html).

**Figure 2:**
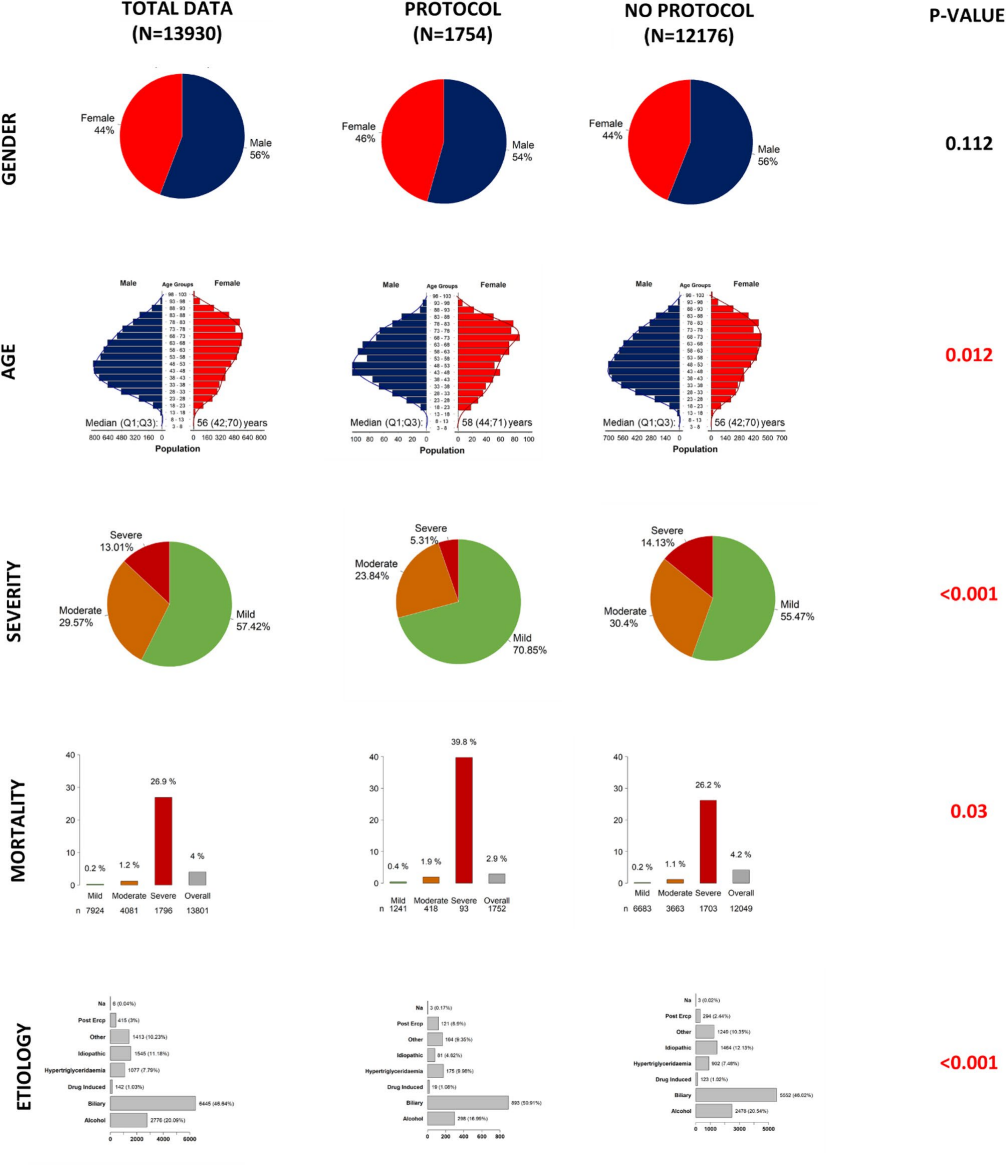
General characteristics of the international cohorts. Comparison of the protocol and non-protocol international cohorts. In terms of age, distribution of severity and etiology, and overall mortality there is a significant difference among the subcohorts (*p* < 0.05).

Overall, 13,930 cases from 3 continents, 23 countries, 56 centres participated in the survey and were analysed. Altogether 1754 (12.59%) cases belong to the international protocol group. The participating countries and the number of uploaded cases are illustrated on a colour-scaled map (Fig. 1) and listed in detail in the supplementary materials (Table S2). The median age was significantly lower in the non-protocol group (58 (Q1;Q3: 44;71) vs. 56 (Q1;Q3: 42;70) years, *p* = 0.012). Furthermore, in the non-protocol group the number of severe cases was significantly higher (14.1% vs. 5.3%) as well as the overall mortality rate (4.2% vs 2.9%, *p* = 0.03) (Fig. 2). Data quality can be seen in Table S4.

### The majority (87.5%) of the international centres have no protocols to discharge patients in AP

According to our international survey, 87.5% (49/56) of the centres did not apply an AP discharge protocol. Notably, the protocols were moderately different from each other. Abdominal pain status was a part of every protocol, but for example, appetite was mentioned only in one case. Further details regarding the elements of discharge protocols are shown in Table S3.

### Protocolized discharge strategy results in shorter length of hospitalization and

Patients discharged based on protocols have significantly shorter length of hospitalization (LOH) (7 (Q1;Q3: 5;10 days) vs. 8 (Q1;Q3: 5;12 days), *p* < 0.001) and lower rate of readmission due to RAP (2.8% vs. 3.9%). When separately analysing the cohorts based on severity, protocolized discharge decision still resulted in significantly shorter LOH both in the mild and moderate/severe cases (10 (Q1;Q3: 7;15 days) vs. 12 (Q1;Q3: 8;18 days)), *p* < 0.001) (Fig. S2).

There was no significant difference in the discharge CRP values between the groups (29.75 (9.26; 80.00) mg/l vs. 28.50 (11.80; 58.40) mg/l, *p* = 0.586) (Table 1). However, when separately analysing the patients based on severity, in the moderate/severe cases the discharge CRP was significantly higher 46.24 (16.65; 100.25) vs. 34.00 (15.70; 59.75) mg/l, *p* = 0.002) (Fig. S2).

**Table 1:** Comparison of centres based on the presence of discharge protocol worldwide and in Hungary.

	International			Hungarian		
	Protocol	No-protocol	*p* values	Protocol	No-protocol	*p* values
Patient number	1754	12,176	NA	688	941	NA
Length of hospitalization						
n (%not missing)	1754 (100)	12,146 (97.8)		688 (100)	920 (97.8)	
mean (SD)	8.55 (8.12)	11.75 (14.30)		8.20 (7.71)	13.04 (15.80)	
median (Q1; Q3)	7 (5; 10.)	8 (5;12)	< 0.001^1^	6 (5; 9)	10 (7;15)	< 0.001^1^
Discharge CRP						
n (%not missing)	1124 (64.1)	8102 (67.2)		688 (100)	482 (51.2)	
mean (SD)	54.31 (61.99)	48.61 (61.95)		48.31 (46.38)	47.41 (59.82)	
median (Q1; Q3)	29.75 (9.26, 80.00)	28.50 (11.80, 58.40)	0.586^1^	35.40 (13.78, 68.40)	22.88 (8.80, 62.03)	0.003^1^
Readmission within 1 month						
n (%not missing)	1727 (98.4)	11,829 (97.2)		688 (100)	609 (64.7)	
readmission n (%)	167 (9.7%)	1101 (9.3%)	0.629^2^	35 (5.09%)	114 (19%)	< 0.001^2^
Not pancreas related	62 (3.6%)	309 (2.6%)	0.005^2^	12 (1.7%)	67 (11%)	< 0.001^2^
Complication of index AP	39 (2.3%)	275 (2.3%)		4 (0.6%)	24 (3.9%)	
Recurrent episode of AP	48 (2.8%)	464 (3.9%)		19 (2.7%)	23 (3.8%)	

^1^Mood’s median test.

^2^Chi-squared test.

The table shows the comparison of centres with and with no discharge protocol, clearly describing that protocolized discharge results in shorter LOH, higher discharge CRP values and lower rate of readmission. LOH is expressed in days, while CRP in mg/l.

### Safety and effectiveness of the HPSG-guided discharge protocol

**Figure 3:**
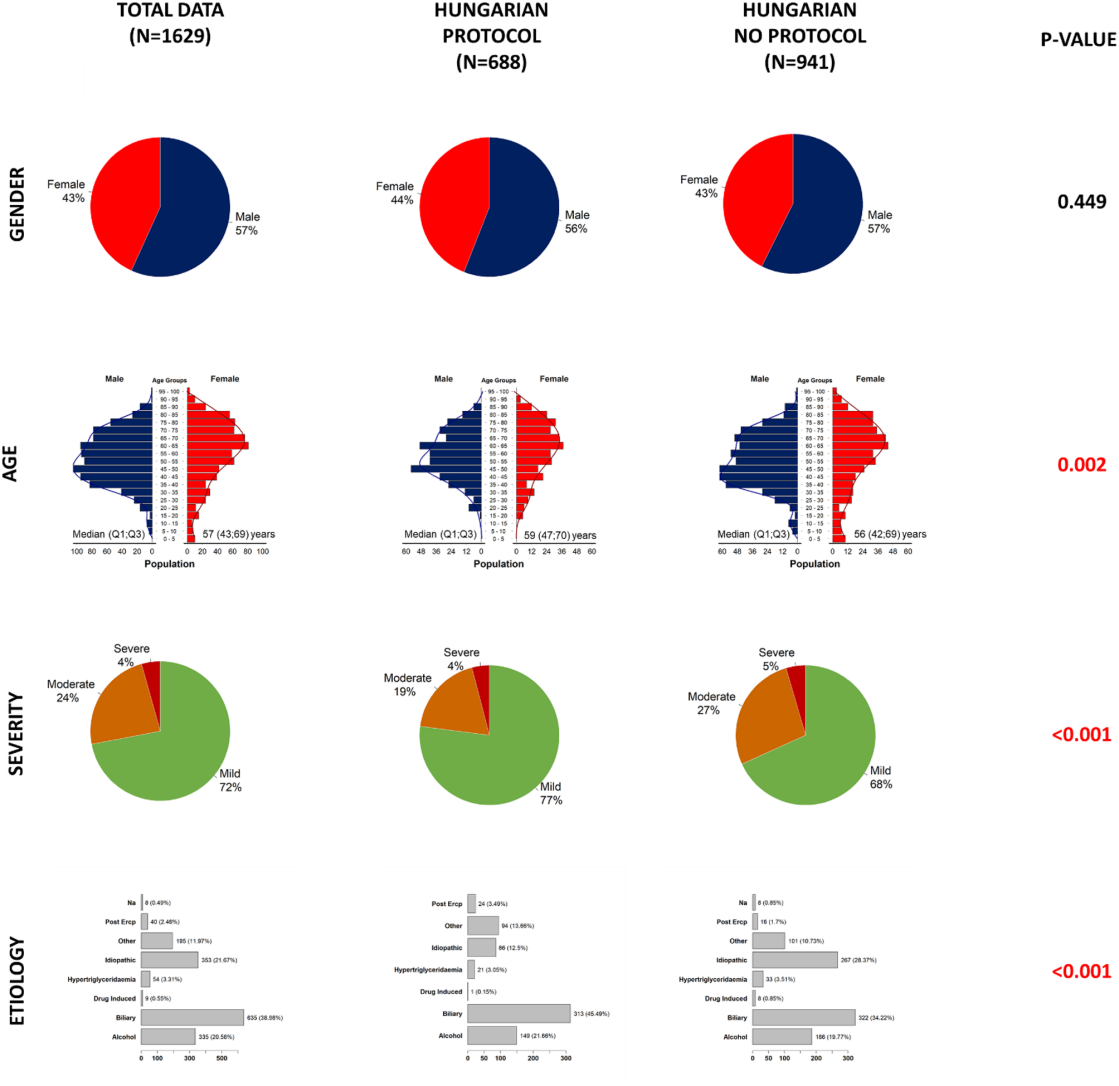
General characteristics of the Hungarian cohorts. Comparison of the protocol and non-protocol Hungarian cohorts. In terms of age, distribution of severity and etiology there is a significant difference among the subcohorts (*p* < 0.05).

Overall, 688 patients were discharged with HPSG-protocol whereas 941 patients without it. The median age of the 2 subcohorts differed in terms of age (median (Q1;Q3): 59 (47;70) vs. (56 (42;69), severity (moderately severe cases: 19% in protocol vs 27% in non-protocol group) and the distribution of the aetiologies (Fig. 3). The median CRP value at discharge was shown to be significantly higher in the HPSG protocol group compared to the non-protocol Hungarian centres (35.40 (13.78; 68.40) vs. 22.88 (8.80; 62.03) mg/l, *p* = 0.003) (Table 1). This remarkable difference was also shown in the mild and moderate/severe cases separately (29.35 (12.22; 59.80 vs. 21.60 (8.33; 60.45) mg/l, *p* = 0.021 and 56.95 (23.17; 95.65) vs. 33.90 (10.55; 71.71), *p* < 0.001, respectively) (Fig S4). We also investigated the death rate within 1 month, based on the data of the Ministry for Home Affairs, 2 patients with serious comorbidities died before the 1-month follow-up visit. Data quality can be seen in Table S4.

#### The HPSG-developed discharge protocol was associated with a lower readmission rate vs non-protocolized discharge (5% vs. 19%)

In order to check the safety of the HPSG-protocol, patients were examined 1 month after discharge. Concerning the protocol-guided discharge, 45 out of 688 patients had elevated CRP value on the 1-month control visit compared to the discharge level (Figure S3). Nine (20%) had biliary tract inflammation (cholangitis, cholecystitis), 14 (31%) had recurrent episode, 6 (13%) had tumour-related complaints, whereas the remaining cases were mostly related non-GI diseases (rheumatoid arthritis, respiratory tract infection; 58%). Out of the 688 patients 35 (5%) were readmitted within 1 month. Among the readmitted patients 19 (54%) had recurrent episode of AP (alcohol induced: 47%, biliary: 26% CP/idiopathic: 26%), 4 (11%) had pseudocyst infection, 4 (11%) had cholecystitis/cholangitis. Five (14%) readmissions occurred due to tumour-related complaints, 2 (6%) other patients had IBD and gastroenteritis, and 1 (3%) was admitted because of trauma. In comparison, in the non-protocolized cohort, 179 of 941 (19%) patient were readmitted, mainly due to non-pancreas-related causes and index episode complication (59% vs. 21%) (Table 2).

**Table 2:** Table of the readmission rates in the HPSG Protocol group.

	Readmitted		Non-readmitted	
	N (%)	discharge CRP*	N (%)	discharge CRP
Overall	35 (5)	51.60 (19.95; 66.45)	653 (95)	35.10 (13.40, 68.70)
Related to AP etiology	19 (54.29)	38.70 (17.95; 57.2)	NA	
Complication of index admission	4 (11.42)	69.15 (66.23; 83.08)	NA	
Other causes	12 (34.29)	55.70 (27.8; 61.20)	NA	
Discharged < 50 mg/l CRP	17 (48.57)	18.60 (12.00; 38.60)	448 (64.1)	17.25 (8.18; 31.43)
Discharged > 50 mg/l CRP	18 (51.43)	66.45 (57.60; 76.60)	205 (35.9)	83.90 (63.35; 112.83)

The table shows the number of readmitted patients due to certain causes. *Values are expressed in median (Q1;Q3). unit: mg/l. NA-not applicable, data are not available. Patients readmitted with pseudocyst infection had the highest median discharge CRP value.

#### Implementation of the new discharge protocol results in shorter hospital stay

One of the most relevant indices concerning the effectiveness is the LOH. Our cohort was shown to have significantly shorter LOH (6 (Q1;Q3: 5;9) days) compared to centres with no protocol either internationally (8 (Q1;Q3: 5;12) days) or nationally (10 (Q1;Q3:7;15) days) (Table 1). The difference in LOH in the Hungarian cohort was shown both in mild and moderate/severe cases when analysed separately (6 (5;7) vs. 9 (7;12.) days) (Fig. S4).

### CRP value proved to be a poor prediction tool

We investigated whether the inflammatory biomarker CRP can predict readmission in AP. Discharge CRP has been identified as a poor prediction tool both in total and only in mild cases for readmission (AUC: 0.56 and 0.56 *p* = ns, respectively) (Fig. S4). In addition, readmission could not be predicted by the rate of decrease after the maximum CRP level (either investigated a 24 or a 48-h period). (*p* = 0.116, 0.208, respectively) (Fig. S5).

## Discussion

In this study we tested the safety and effectiveness of discharge protocols in AP. We found that protocols significantly decrease the LOH and do not elevate the risk of readmissions. Protocolized discharge also resulted in higher discharge CRP values that may suggest, physicians are more confident in making discharge decisions in the presence of a protocol-based care.

Sheila Serra et al. showed that discharge patients with mild AP within 48 h is safe if the CRP level is below 15 mg/dl, the blood urea nitrogen change in 24 h interval is below 5 mg/dl and they tolerate oral intake^11^. An Australian study examined the possible risk factors which can lead to justified longer LOH than 2 days. Higher body temperature (> 38 °C), not tolerating oral diet by day 2, high pain score (VAS > 5), and high white blood cell level (> 18 G/L) were identified as risk factors. However, 87% of the admitted patients with mild AP could have been discharged at day 2 and transferred to outpatient clinic^14^. All these findings raise the question whether the vast majority of the patients do not require several days of hospitalization but an intensive outpatient follow-up. In other diseases, there were also positive results from the mindful patient discharge. Naureen et al. implemented a standardized, evidence-based discharge protocol when discharging patients with heart failure and consequently, it was shown that patient education can positively impact self-management after discharge resulting in shorter LOH and lower 30-day readmission rates^15^.

According to our results the protocol follower centres were identified to have lower 1-month readmission rate. This finding can be explained by the fact that these institutions most probably apply a strict etiology workup and follow additional AP-related guidelines, such as on-admission cholecystectomy or implement efficient patient education, thus lowering the number of recurrent or even the severe cases^16,17^. Furthermore, Whitlock at el. built up a model in which treatment with antibiotics, pain, pancreas necrosis, and gastrointestinal symptoms were identified as a risk factor for early (within 30 days) readmission^18^.

The proportion of severe cases in the non-protocol group is markedly higher, especially in the international cohort, despite the fact that it can be assumed that protocolized institutions operate as tertiary centres where a relatively large number of severe cases are transferred. However, we need to mention that since there is a higher proportion of moderate or severe cases in the non-protocol groups requiring antibiotic treatment, and having local or systemic complications, it could also contribute to the longer LOH and lower CRP level at discharge.

Of course, the prediction of possible readmission is of utmost importance and, therefore, we investigated whether CRP could be a reliable predictive tool. Unfortunately, CRP failed to be useful in this situation. CRP level as a prediction tool for readmission at discharge was investigated in several fields but not in AP. Acute heart failure patients discharged with elevated CRP value (> 10 mg/L) value, were shown to have a higher risk of mortality and readmission^19,20^. Furthermore, investigation of the delayed complications after esophagectomy showed that discharge patients with CRP level < 84 mg/L on day 7 proved to be a safe approach, however CRP trend itself could not predict delayed complications^21^. In our cohort, neither the absolute CRP value nor the degree of the decline showed a significant relationship with the 1-month readmission, supporting the theory that patient discharge should not depend on the current value or the volume of the decrease but rather on the direction of the tendency.

### Strengths and limitations

The strength of our study is that we conducted an international survey including 23 countries from 3 continents and extensive data were collected. The data quality, especially in the HPSG registry analysis is remarkably high. However, we have to mention the limitations, such as the retrospective nature of the international cohort analysis. There is no information about the number of tertiary centres in our analysis, which can highly influence the number and characteristics of admitted patients. In the Hungarian cohort analyses, there might be slight differences in the way how the HPSG-discharge protocol was applied in different centres. Furthermore, the fact that those centres that apply protocols probably provide better patient care anyway.

### Implication for practice and research

Implementing scientific data in the daily practice have high importance (6,7). The HPSG-discharge protocol can be immediately used in practice. Following an evidence-based discharge protocol will result in shorter LOH and thus, lower costs and also lower risk of hospital acquired infections. Is this the best possible protocol to implement? Probably not, therefore new protocols are warranted. Importantly, when additional individualized discharge protocols are possible, the individualized solution may lead to even better results. For the better assessment, randomized clinical trials are needed to be performed.

## Conclusion

Using discharge protocols in AP shorten the hospital stay. The HPSG-protocol resulted in the shortest LOH and still did not increase the risk of readmittance. There is a particular need for evidence-based recommendations on discharge in guidelines.

### Supplementary Information

Supplementary Information.

## Data Availability

The datasets generated during and/or analysed during the current study are available from the corresponding author on reasonable request.
