# Supplementary material for: Discharge protocol in acute pancreatitis: an international survey and cohort analysis
Source: Sci Rep. 2023 Dec 13;13:22109. doi: 10.1038/s41598-023-48480-z (PMC10719286; doi:10.1038/s41598-023-48480-z)
Supplement: Supplementary file 1 — Supplementary Information. [file 41598_2023_48480_MOESM1_ESM.docx]

**Nagy et al., Discharge protocol in acute pancreatitis: an international survey and cohort analysis**

SUPPLEMENTARY MATERIAL

Rita Nagy^1,2,3^, Klementina Ocskay^3^, Zoltán Sipos^2^, Andrea Szentesi^2^, Áron Vincze^4^, László Czakó^5^, Ferenc Izbéki^6^, Natalia V. Shirinskaya^7^, Vladimir L. Poluektov^8^, Alexandr N. Zolotov^9^, Yin Zhu^10^, Liang Xia^10^, Wenhua He^10^, Robert Sutton^11^, Peter Szatmary^11^, Rajarshi Mukherjee^11^, Isobel Saffron Burridge^12^, Emma Wauchope^12^, Elsa Francisco^13^, David Aparicio^13^, Bruno Pinto^13^, António Gomes^13^, Vitor Nunes^13^, Vasile Marcel Tantau^14^, Emanuela Denisa Sagau^14^, Alina Ioana Tantau^15^, Andra Iulia Suceveanu^16^, Cristina Tocia^16^, Andrei Dumitru^16^, Elizabeth Pando^17^, Piero Alberti^17^, Arturo Cirera^17^, Xavier Molero^18^, Hong Sik Lee^19^, Min Kyu Jung^19^, Eui Joo Kim^20^, Sanghyub Lee^21^, María Lourdes Ruiz Rebollo^22^, Reyes Busta Nistal^22^, Sandra Izquierdo Santervas^22^, Dusan Lesko^23^, Marek Soltes^23^, Jozef Radonak^23^, Hubert Zatorski^24^, Ewa Małecka-Panas^24^, Adam Fabisiak^24^, Susak Yaroslav M.^25^, Maksymenko Mykhailo V.^25^, Tkachenko Olekcandr A.^26^, Giedrius Barauskas^27^, Vytautas Simanaitis^27^, Povilas Ignatavicius^26^, Mariana Jinga^27^, Vasile-Daniel Balaban^27^, Cristina Patoni^28^, Liang Gong^29^, Kai Song^29^, Yunlong Li^29^, Cúrdia Gonçalves T.^31,32,33^, Marta Freitas^30^, Vítor Macedo^30,31,32^, Marlies Vornhuelz ^34^, Sarah Klauss^34^, Georg Beyer^34^, Aydin Seref Koksal^35^, Mukaddes Tozlu^35^, Ahmet Tarik Eminler^35^, Nuria Torres Monclús^36^, Eva Pijoan Comas^36^, Juan Armando Rodriguez Oballe^36^, Łukasz Nawacki^37^, Stanisław Głuszek^37^, Alberto Rama-Fernández^38^, Marco Galego^38^, Daniel de la Iglesia^38^, Umut Emre Aykut^39^, Deniz Güney Duman^39^, Rahmi Aslan^39^, Adriana Gherbon^40^, Lihui Deng^41^, Wei Huang^41^, Qing Xia^41^, Goran Poropat^42^, Anja Radovan^42^, Luka Vranić^42^, Claudio Ricci^43,44^, Carlo Ingaldi^43,44^, Riccardo Casadei^43,44^, Ionut Negoi^45^, Cezar Ciubotaru^45^, Florin Mihail Iordache^45^, Gabriel Constantinescu^45^, Vasile Sandru^45^, Engin Altintas^46^, Hatice Rizaoglu Balci^46^, Júlio Constantino^47^, Débora Aveiro^47^, Jorge Pereira^47^, Suleyman Gunay^48^, Seda Misirlioglu Sucan^48^, Oleksiy Dronov^49^, Inna Kovalska^49^, Nikhil Bush^50^, Surinder Singh Rana^50^, Serge Chooklin^51^, Serhii Chuklin^51^, Ionut Adrian Saizu^52^, Cristian Gheorghe^29,52^, Philipp Göltl^53^, Michael Hirth^53^, Radu Bogdan Mateescu^29,54^, Geanina Papuc^54^, Georgi Angelov Minkov^55^, Emil Tihomirov Enchev^55^, Laura Mastrangelo^56^, Elio Jovine^56^, Weiwei Chen^57^, Quping Zhu^57^, Anita Gąsiorowska^58^, Natalia Fabisiak^58^, Mihailo Bezmarevic^59^, Andrey Litvin^60^Martina Cattani Mottes^61^, Eun Kwang Choi^62^, Peter Bánovčin^63^, Lenka Nosáková^63^, Mila Dimitrova Kovacheva-Slavova^64^, Ali Kchaou^65^, Ahmed Tlili^66^, Marco V. Marino^67^, Katarzyna Kusnierz^68^, Artautas Mickevicius^68^, Marcus Hollenbach^69^, Pavol Molcan^70^, Orestis Ioannidis^71^, Mark Valerievich Tokarev^72^, Ali Tüzün Ince^73^, Ivan Albertovich Semenenko^74^, Shamil Galeev^75^, Elena Ramírez-Maldonado^76^, Ville Sallinen^77^, Petr Pencik^78^, Judit Bajor^4^, Patricia Sarlós^4^, Roland Hágendorn^4^, Szilárd Gódi^4^, Imre Szabó^4^, József Czimmer^4^, Gabriella Pár^4^, Anita Illés^4^, Nándor Faluhelyi^79^, Péter Kanizsai^80^, Tamás Nagy^81^, Alexandra Mikó^2^, Balázs Németh^5^, József Hamvas^82^, Barnabás Bod^83^, Márta Varga^84^, Imola Török^85^, János Novák^86^, Árpád Patai^87^, János Sümegi^88^, Csaba Góg^89^, Mária Papp^90^, Bálint Erőss^2,91^, Szilárd Váncsa^2,91,92^, Brigitta Teutsch^2,92^, Katalin Márta^91^, Péter Jenő Hegyi^91^, Tamás Tornai^91^, Balázs Lázár^91^, Tamás Hussein^91^, Dorottya Tarján^91^, Mónika Lipp^91^, Beáta Kovács^91^, Orsolya Urbán^91^, Emese Fürst^91^, Edina Tari^91^, Ibolya Kocsis^92^, Pál Maurovich-Horvát^93^, Balázs Tihanyi^94^, Orsolya Eperjesi^91^, Zita Kormos^91^, Pál Ákos Deák^95^, Andrea Párniczky ^1,2,3^ and Péter Hegyi^1,2,91,96*^, on behalf of the Hungarian Pancreatic Study Group and the Collaborating Study Group

^1^ Centre for Translational Medicine, Semmelweis University, Budapest, Hungary

^2^ Institute for Translational Medicine, Medical School, University of Pécs, Pécs, Hungary

^3^ Heim Pál National Pediatric Institute, Budapest, Hungary

^4^Division of Gastroenterology, First Department of Medicine, Medical School, University of Pécs, Pécs, Hungary

^5^Department of Medicine, University of Szeged, Szeged, Hungary

^6^Szent György University Teaching Hospital of Fejér County, Székesfehérvár, Hungary

^7^Omsk State Medical University, Omsk State Medical Information-Analytical Centre, Omsk, Russia

^8^Department of Surgery and Urology, Omsk State Medical University, Omsk, Russia

^9^Department of Pathophysiology, Clinical Pathophysiology, Omsk State Medical University, Omsk, Russia

^10^Department of Gastroenterology, First Affiliated Hospital of Nanchang University, Nanchang, China

^11^Unversity of Liverpool, Liverpool University Hospitals NHS Foundation Trust, Liverpool, United Kingdom

^12^Liverpool University Hospitals NHS Foundation Trust, Liverpool, United Kingdom

^13^Surgery Department, Hospital Prof. Ferndo Fonseca, Amadora, Portugal

^14^ ”Octavin Fodor”Institute of Gastroenterology and Hepartology, ”Iuliu Hatieganu” University of Medicine and Pharmacy, Cluj Napoca, Romania

^15^Gastroenterology Department, 4th Medical Clinic,”Iuliu Hatieganu” University of Medicine and Pharmacy, Cluj Napoca, Romania

^16^Faculty of Medicine, Ovidius University of Constanta, Constanta, Romania

^17^Department of Hepato-Pancreato-Biliary and Transplant Surgery, Hospital Universitari Vall d'Hebron, Universitat Autònoma de Barcelona, Barcelona, Spain

^18^Exocrine Pancreas Research Unit, Hospital Universitari Vall d'Hebron, Institut de Recerca, Universitat Autònoma de Barcelona, CIBEREHD, Barcelona, Spain

^19^Division of Gastroenterology and Hepatology, Department of Internal Medicine, Korea University Anam Hospital, Seoul,Republic of Korea

^20^Division of Gastroenterology, Department of Internal Medicine, Gachon University Gil Medical Center, Gachon University College of Medicine, Incheon, Republic of Korea

^21^Department of Internal Medicine and Liver Research Institute, Seoul National University Hospital, Seoul, Republic of Korea

^22^Servicio de Aparato Digestivo Hospital Clínico Universitario Valladolid, Valladolid, Spain

^23^1st Department of Surgery, University Hospital of L.Pasteur, Kosice, Slovak Republic

^24^Department of Digestive Tract Diseases, Medical University of Lodz, Lodz, Poland

^25^Department of Surgery with a Course of Emergency and Vascular Surgery, Bogomolet National medical University, Kiev, Ukraine

^26^Kyiv City Clinical Emergency Hospital, Kiev, Ukraine

^27^Department of Surgery, Lithuanian University of Health Sciences, Kaunas, Lithuania

^28^; "Carol Davila" University of Medicine and Pharmacy, Bucharest, Romania

^29^University of Medicine and Pharmacy "Carol Davila", Bucharest, Romania

^30^Department of Gastroenterology, Peking Union Medical College Hospital, Chinese Academy of Medical Sciences & Peking Union Medical College, Beijing, China

^31^Gastroenterology Department, Hospital da Senhora da Oliveira, Guimarães, Portugal

^32^ Life and Health Sciences Research Institute (ICVS), School of Medicine, University of Minho, Braga/ Guimarães, Portugal

^33^ICVS/3B’s–PT Government Associate Laboratory, Braga/ Guimarães, Portugal

^34^ LMU University Hospital, LMU Munich, Germany

^35^Department of Gastroenterology, Sakarya University, Faculty of Medicine, Sakarya, Turkey

^36^University Hospital Arnau de Vilanova, Hospital University Santa Maria, Lleida, Spain

^37^Collegium Medicum, The Jan Kochanowski University in Kielce, Kielce, Poland

^38^Gastroenterology Department, University Hospital of Santiago de Compostela, Santiago de Compostela, Spain

^39^Marmara University Education and Training Hospital, Istanbul, Turkey

^40^Discipline of Internal Medicine: Diabetes, Nutrition, Metabolic Diseases and Systemic Rheumatology, Victor Babeș University of Medicine and Pharmacy Timisoara, Romania

^41^Department of Integrated Traditional Chinese and Western Medicine, Sichuan Provincial Pancreatitis Center and West China-Liverpool Biomedical Research Center, West China Hospital, Sichuan University, Chengdu, China

^42^Department of Gastroenterology, Clinical Hospital Center Rijeka, University of Rijeka, Rijeka, Croatia

^43^Division of Pancreatic Surgery, IRCCS, Azienda Ospedaliero Universitaria di Bologna, Bologna, Italy

^44^Department of Internal Medicine and Surgery (DIMEC), Alma Mater Studiorum, University of Bologna, S.Orsola-Malpighi Hospital, Bologna, Italy

^45^Carol Davila University of Medicine and Pharmacy Bucharest, Emergency Hospital of Bucharest, Bucharest, Romania

^46^Gastroenterology Department, Mersin University, Faculty of Medicine, Yenisehir/Mersin, Turkey

^47^Unidade HBP, Serviço de Cirurgia Geral, Centro Hospitalar Tondela-Viseu, Viseu, Portugal

^48^İzmir Katip Çelebi University Atatürk Training and Research Hospital, Karabaglar/Izmir, Turkey

^49^General Surgery #1, Bogomolets National Medical University, Kiev, Ukraine

^50^Department of Gastroenterology, Postgraduate Institute of Medical Education and Research (PGIMER), Chandigarh, India

^51^Lviv Regional Clinical Hospital, Lviv, Ukraine

^52^Clinical Institute Fundeni, Bucharest, Romania

^53^Department of Medicine II, University Medical Center Mannheim, Medical Faculty Mannheim, Heidelberg University, Mannheim, Germany

^54^Gastroenterology Department, Colentina Clinical Hospital Bucharest, Bucharest, Romania

^55^Department of Surgery, University Hospital, Stara Zagora, Bulgaria

^56^Department of Surgery, AOU Sant’Orsola Malpighi, IRCCS Azienda Ospedaliera Universitaria , Bologna , Italy

^57^Department of Gastroenterology, Clinical Medical College, Yangzhou University, Yangzhou, Jiangsu, China

^58^Department of Gastroenterology Medical University of Lodz, Lodz, Poland

^59^Department for Hepatobiliary and Pancreatic Surgery, Clinic for General Surgery, Military Medical Academy, University of Defense, Belgrade, Serbia

^60^Gomel State Medical University, Belarus

^61^Department of Medicine, Gastroenterology, The Pancreas Institute, G.B. Rossi University Hospital, Verona, Italy

^62^Department of Internal Medicine, Jeju National University College of Medicine, Jeju, South Korea

^63^ Clinic of internal medicine - gastroenterology, JFM CU, Jessenius Faculty of Medicine in Martin (JFM CU), Comenius University in Bratislava, Slovakia

^64^Department of Gastroenterology, Queen Yoanna University Hospital, Medical University of Sofia, Sofia, Bulgaria

^65^Habib Bourguiba University Hospital, Sfax, Tunisia

^66^Mohamed Ben Sassi Hospital, Gabes, Tunisia

^67^General Surgery Department, Azienda Ospedaliera Ospedali Riuniti Villa Sofia-Cervello, Palermo, Italy

^68^Vilnius University Hospital Santariskiu Klinikos, Vilnius, Lithuania

^69^Division of Gastroenterology, University of Leipzig Medical Center, Leipzig, Germany

^70^Hepatology and Gastroenterology Departement of Roosvelt Hospital, Banska Bystrica, Slovakia

^71^4th Department of Surgery, Medical School, Aristotle University of Thessaloniki, General Hospital “George Papanikolaou”, Thessaloniki, Greece

^72^Sklifosovsky Institute for Clinical Medicine, Sechenov First Moscow State Medical University, Moscow, Russia

^73^Hospital of Bezmialem Vakif University, School of Medicine, Istanbul, Turkey

^74^Sechenov University, Moscow, Russia

^75^Saint Luke Clinical Hospital, St. Petersburg, Russia

^76^General Surgery, Consorci Sanitari del Garraf, Sant Pere de Ribes, Barcelona, Spain

^77^Department of Transplantation and Liver Surgery, Helsinki University Hospital and University of Helsinki, Helsinki, Finland

^78^Centrum péče o zažívací trakt, Vítkovická nemocnice a.s., Ostrava, Czech Republic

^79^Department of Medical Imaging, Medical School, University of Pécs, Pécs, Hungary

^80^Department of Emergency Medicine, Medical School, University of Pécs, Pécs, Hungary

^81^Department of Laboratory Medicine, Medical School, University of Pécs, Pécs, Hungary

^82^Peterfy Hospital, Budapest, Hungary

^83^Dr. Bugyi István Hospital, Szentes, Hungary

^84^Department of Gastroenterology, BMKK dr Rethy Pal Hospital, Békéscsaba, Hungary

^85^County Emergency Clinical Hospital of Târgu Mures - Gastroenterology Clinic and University of Medicine, Pharmacy, Sciences and Technology ”George Emil Palade”, Targu Mures, Romania

^86^Pándy Kálmán Hospital of Békés County, Gyula, Hungary

^87^Markusovszky University Teaching Hospital, Szombathely, Hungary

^88^Borsod-Abaúj-Zemplén County Hospital and University Teaching Hospital, Miskolc, Hungary

^89^Healthcare Center of County Csongrád, Makó, Hungary

^90^Department of Gastroenterology, Institute of Internal Medicine, Faculty of Medicine, University of Debrecen, Debrecen, Hungary

^91^Institute of Pancreatic Diseases, Semmelweis University, Budapest, Hungary

^92^Department of Laboratory Medicine, Semmelweis University, Budapest, Hungary

^93^MTA-SE Cardiovascular Imaging Research Group, Medical Imaging Centre, Semmelweis University, Budapest, Hungary

^94^ Department for Surgery, Hungarian Defence Forces - Medical Centre, Budapest, Hungary.

^95^Medical Imaging Centre, Department of Radiology, Semmelweis University, Budapest, Hungary

^96^ Translational Pancreatology Research Group, Interdisciplinary Centre of Excellence for Research Development and Innovation University of Szeged, Szeged, Hungary

**Content**

[FIGURE S1. The online questionnaire used in the international survey 5](#_Toc131420453)

[TABLE S1. Excel table for data collection 6](#_Toc131420454)

[TABLE S2. Table of the institutions participating in the analysis 7](#_Toc131420455)

[TABLE S3. Table of reported discharge protocols 12](#_Toc131420456)

[FIGURE S2. Length of hospitalisation and discharge CRP values based on severity visualized by boxplots 13](#_Toc131420457)

[FIGURE S3. Line chart showing the change of CRP level after discharge until 1-month visit 14](#_Toc131420458)

[TABLE S4. Data quality 15](#_Toc131420459)

[FIGURE S4. ROC curve and AUC value representing the sensitivity and specificity of discharge CRP level in terms of readmission in all (a.) and only in mild AP cases (b.). 16](#_Toc131420460)

[FIGURE S5. Relationship of 24 and 48 hours decreasing tendency in CRP and readmisson rates 17](#_Toc131420462)

# FIGURE S1. The online questionnaire used in the international survey


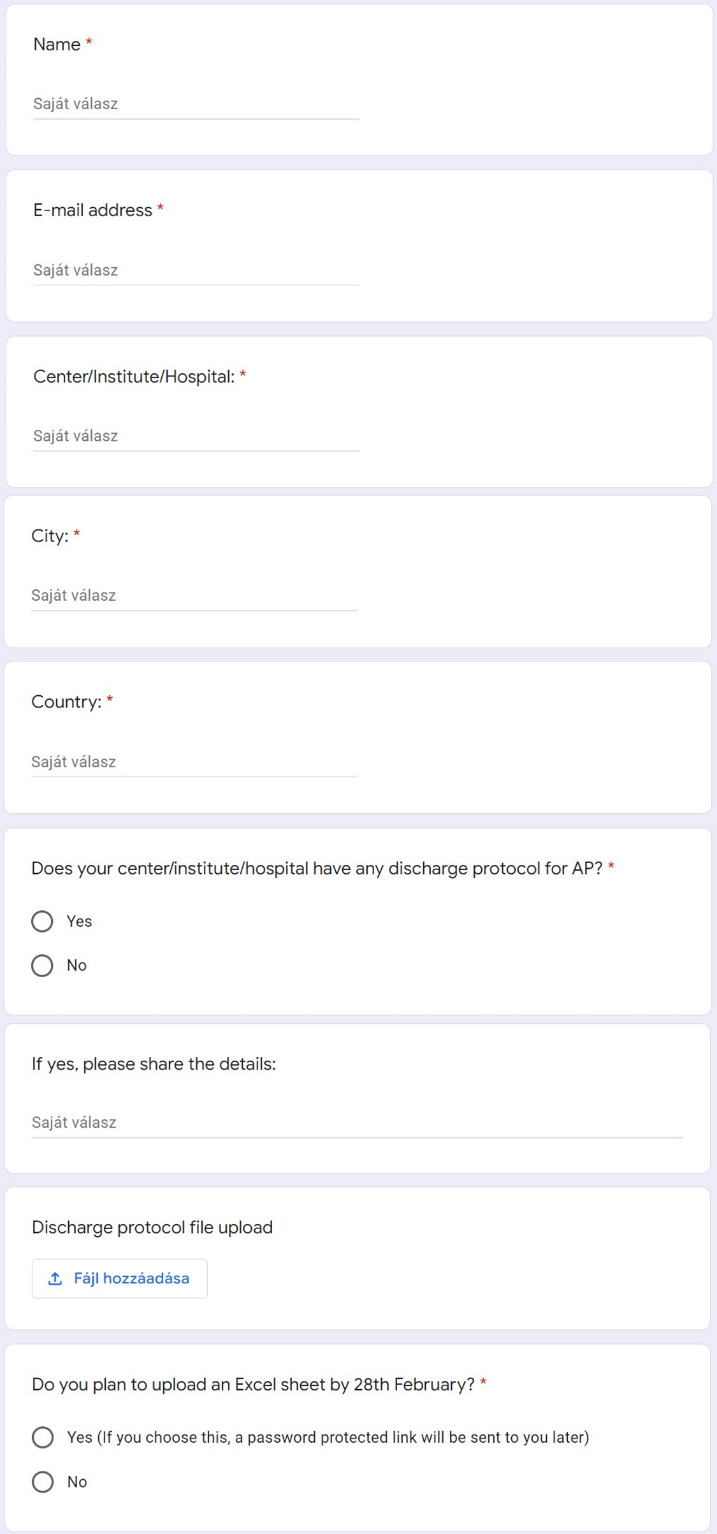


Online sheet used for the international survey where the centres were asked to provide information if they apply a discharge protocol and if yes, give its elements.

g

# TABLE S1. Excel table for data collection

## TABLE S2. Table of the institutions participating in the analysis

|  | **Country** | **Center** | **Protocol** | **Patient number** | **Length of hospitalization** | | **Discharge CRP** | | **1-month readmission rate** | |
| --- | --- | --- | --- | --- | --- | --- | --- | --- | --- | --- |
|  |  |  |  |  | **mean ± SD** | **median (Q1, Q3)** | **mean ± SD** | **median (Q1, Q3)** | **n** | **%** |
| 1 | Bulgaria | Department of Gastroenterology, Queen Yoanna University Hospital, Medical University of Sofia | NO | 60 | 9.23   ±  6.76 | 7   (4, 11) | 84.89   ±  115.59 | 17.5   (4.65, 137.2) | 0 | 0% |
| 2 | Bulgaria | University Hospital, Department of Surgery, Stara Zagora | NO | 116 | 11.47   ±  7.24 | 9   (7, 14) | 68.1   ±  69.8 | 54.45   (23.45, 86.93) | 21 | 18.10% |
| 3 | China | Department of Gastroenterology, Clinical Medical College, Yangzhou University | NO | 106 | 6.75   ±  2.31 | 6   (5, 8) | 39.81   ±  58.86 | 19.95   (6.55, 48.94) | NA | NA |
| 4 | China | Department of Gastroenterology, First Affiliated Hospital of Nanchang University, Nanchang | NO | 1096 | 12.18   ±  13.83 | 8   (5, 13) | 57.01   ±  70.39 | 25.4   (10.6, 84.45) | 109 | 9.95% |
| 5 | China | Department of Gastroenterology, Peking Union Medical College Hospital | NO | 306 | 20.86   ±  16.47 | 16   (10, 27) | 31.7   ±  40.15 | 15   (5.5, 41.5) | 50 | 16.34% |
| 6 | China | West China Hospital, Sichuan University | NO | 216 | 10.31   ±  6.63 | 9   (6, 13) | 57.88   ±  86.2 | 19.7   (6.86, 78.1) | 1 | 0.46% |
| 7 | Croatia | Department of Gastroenterology, Clinical Hospital Center Rijeka, University of Rijeka | NO | 202 | 10.71   ±  8.04 | 9   (6.25, 12.75) | 40.11   ±  53.3 | 18.2   (8.1, 54.5) | 10 | 4.95% |
| 8 | Czech Republic | Centrum péče o zažívací trakt, Vítkovická nemocnice a.s., Ostrava | NO | 11 | 8.18   ±  2.44 | 8   (6.5, 10) | 43.47   ±  52.05 | 20.1   (9.5, 58.3) | NA | NA |
| 9 | Finland | Helsinki University Central Hospital | NO | 25 | 4.08   ±  2.77 | 3   (3, 4) | 101.24   ±  78.77 | 84   (64, 113) | NA | NA |
| 10 | Germany | Department of Medicine II, University Medical Center Mannheim, Heidelberg University, Mannheim | NO | 126 | 7.81   ±  10.9 | 6   (4, 8) | 62.95   ±  62.32 | 48.3   (14, 93.25) | 14 | 11.11% |
| 11 | Germany | Department of Internal Medicine II, LMU Hospital, Munich | NO | 295 | 9.94   ±  11.28 | 7   (4, 11) | 50.87   ±  59.19 | 28   (12, 69) | 24 | 8.14% |
| 12 | Germany | Division of Gastroenterology, University of Leipzig Medical Center | NO | 26 | 15.35   ±  16.84 | 8   (5, 20.25) | 24.73   ±  19.8 | 19   (9.75, 30.75) | 4 | 15.38% |
| 13 | Greece | 4th Deparment of Surgery, School of Medicine, Aristotle University of Thessaloniki | NO | 24 | 12.04   ±  11.31 | 8   (7, 11.25) | 4.3   ±  7.95 | 1.27   (0.97, 1.89) | 3 | 12.50% |
| 14 | Hungary | Békés County Central Hospital, Dr. Réthy Pál Hospital, Békéscsaba | NO | 84 | 16.14   ±  8.68 | 14   (10, 19) | 27.93   ±  33.06 | 12.96   (5.43, 43.33) | NA | NA |
| 15 | Hungary | BAZ Central County Central Hospital | NO | 16 | 13.12   ±  5.9 | 13   (10.75, 14.5) | 56.8   ±  56.28 | 42   (9.05, 95.46) | NA | NA |
| 16 | Hungary | Bajcsy-Zsilinszky Hospital, Budapest | NO | 159 | 13.39   ±  12.56 | 10   (8, 15) | 59.34   ±  62.38 | 40.7   (10.45, 76.85) | NA | NA |
| 17 | Hungary | Dr. Bugyi Istvan Hospital, Szentes | NO | 85 | 10.70 ± 8.43 | 9 (7, 13) | 41.14 ± 71.71 | 16.50 (7.5, 37.4) | 11 | 12.94% |
| 18 | Hungary | Department of Emergency, University of Szeged | NO | 10 | 10.68   ±  8.05 | 11 (7, 13) | 22.92 ± 22.57 | 13.55 (12.42, 20.45) | 2 | 20.00% |
| 19 | Hungary | 3rd Int. Medicine/Gastroenterology, Pándy Kálmán Hospital, Gyula | NO | 41 | 10.53   ±  5.85 | 8.5   (7, 11.5) | 49.06   ±  42.83 | 33   (13.53, 77.01) | NA | NA |
| 20 | Hungary | Heim Pál National Pediatric Hospital, Budapest | NO | 28 | 10.17   ±  4.89 | 11   (7.25, 13) | 10.63   ±  12.31 | 5   (0.9, 18.48) | NA | NA |
| 21 | Hungary | Bács-Kiskun County Hospital, Kecskemét | NO | 18 | 14.72   ±  12.98 | 13   (8.25, 17.5) | 54.93   ±  98.41 | 15.65   (12.16, 29.95) | NA | NA |
| 22 | Hungary | Csongrad County Health Center,Makó-Hódmezővásárhely | NO | 10 | 25.1   ±  13.78 | 21.5   (16.25, 31.25) | 10.39   ±  11.3 | 6.23   (2.74, 15.74) | NA | NA |
| 23 | Hungary | 1st Department of Pediatrics, Semmelweis University, Budapest | NO | 12 | 16.18   ±  7.49 | 14   (9.5, 20.5) | 0.1   ±  0.14 | 0.1   (0.05, 0.15) | NA | NA |
| 24 | Hungary | Ist Dep. of Internal Medicine, Szeged | NO | 367 | 12.78   ±  21.77 | 9   (6, 14) | 49.55   ±  61.88 | 29.35   (10.65, 60.25) | NA | NA |
| 25 | Hungary | II. Hospital, Szeged | NO | 46 | 11.02   ±  8.38 | 9   (6.25, 12) | 60.19   ±  52.41 | 59.1   (12.35, 95.5) | NA | NA |
| 26 | Hungary | 2nd Dep. of Internal Medicine, Szeged | NO | 36 | 12.39   ±  8.98 | 12   (8.75, 14) | 31.88   ±  47.4 | 12   (5.1, 34.3) | NA | NA |
| 27 | Hungary | Markusovszky University Teaching Hospital, Szombathely | NO | 29 | 15.52   ±  8.85 | 12   (11, 16) | 33.36   ±  50.3 | 14.6   (7.5, 40.9) | NA | NA |
| 28 | Hungary | University of Debrecen | YES | 8 | 8.88   ±  3.44 | 9.5   (7.25, 11.25) | 42.73   ±  58.14 | 23   (15.51, 40.38) | NA | NA |
| 29 | Hungary | Saint George University Teaching Hospital of County-Fejér | YES | 140 | 7.76   ±  4.39 | 6.5   (5, 9) | 52.62   ±  46.5 | 39.25   (13.6, 83.93) | 5 | 3.57% |
| 30 | Hungary | University of Pécs | YES | 540 | 8.31   ±  8.4 | 6   (5, 8) | 47.28   ±  46.19 | 35.2   (13.88, 64.5) | 30 | 5.56% |
| 31 | India | Postgraduate Institute of Medical Education and Research (PGIMER), Chandigarh | NO | 145 | 21.57   ±  17.35 | 15   (9, 30) | NaN   ±  NA | NA   (NA, NA) | 12 | 8.28% |
| 32 | Italy | Department of Gastroenterology , The Pancreas Institute, G.B. Rossi University Hospital, Verona | NO | 82 | 35.17   ±  59.44 | 16.5   (9.25, 40.75) | 35.32   ±  45.83 | 16   (6.75, 40.75) | 3 | 3.66% |
| 33 | Italy | Department of Surgery, AOU Sant’Orsola Malpighi, IRCCS Azienda Ospedaliera Universitaria, Bologna | NO | 111 | 11.93   ±  16.6 | 9   (6, 13) | 50.34   ±  58.12 | 25.15   (9.4, 92) | 10 | 9.01% |
| 34 | Italy | General Surgery Dpt., Azienda Ospedaliera Ospedali Riuniti Villa Sofia-Cervello, Palermo | NO | 35 | 6.74   ±  1.36 | 7   (6, 8) | 2.51   ±  2.06 | 1.7   (1.5, 2.2) | 5 | 14.29% |
| 35 | Italy | S. Orsola-Malpighi Hospital, University of Bologna | NO | 202 | 11.82   ±  10.79 | 9   (6, 14) | 41.3   ±  57.58 | 17.25   (6.57, 54.52) | 22 | 10.89% |
| 36 | Lithuania | Lithuanian University of Health Sciences, Kaunas | NO | 324 | 15.73   ±  16.83 | 11   (7, 17) | 51.15   ±  81.28 | 19   (8, 48) | 13 | 4.01% |
| 37 | Lithuania | Vilnius, Vilnius University Hospital Santariskiu Klinikos (Santariškių Klinikos) | NO | 31 | 10.61   ±  4.02 | 10   (8, 12) | 24   ±  27.4 | 13.05   (9.85, 28.38) | NA | NA |
| 38 | Poland | Collegium Medicum, The Jan Kochanowski University in Kielce | NO | 260 | 7.52   ±  7.74 | 5   (5, 7) | 119.37   ±  107.16 | 79.21   (28.4, 193.97) | 12 | 4.62% |
| 39 | Poland | Department of Gastroenterology, Medical University of Lodz | NO | 105 | 9.55   ±  4.35 | 9   (7, 11) | 61.56   ±  81.93 | 21.2   (5.8, 95.3) | 7 | 6.67% |
| 40 | Poland | Department of Gastrointestinal Surgery, Medical University of Silesia ,Katowice | NO | 32 | 32.84   ±  23.15 | 29   (17.25, 40) | 112.79   ±  116.03 | 105.1   (56.1, 139) | 3 | 9.38% |
| 41 | Poland | Medical University of Lodz | NO | 330 | 10.9   ±  6.65 | 9   (7, 13) | 39.24   ±  41.19 | 22   (10.7, 56.5) | 12 | 3.64% |
| 42 | Portugal | Gastroenterology Department, Hospital da Senhora da Oliveira - Guimarães | NO | 301 | 9.64   ±  13.1 | 6   (5, 10) | 46.06   ±  58.88 | 25.2   (7.7, 63.9) | 37 | 12.29% |
| 43 | Portugal | Hospital Prof. Ferndo Fonseca, Amadora | NO | 541 | 11.31   ±  13.64 | 7.58   (4.72, 12.31) | 49.06   ±  67.92 | 24.85   (8.87, 58.3) | 51 | 9.43% |
| 44 | Portugal | Unidade HBP, Serviço de Cirurgia Geral, Centro Hospitalar Tondela-Viseu, Viseu | NO | 201 | 10.43   ±  15.58 | 8   (5, 11) | 53.87   ±  71.46 | 31.25   (13.4, 57.33) | 13 | 6.47% |
| 45 | Romania | ”Octavin Fodor”Institute of Gastroenterology and Hepartology, Cluj Napoca | YES | 491 | 8.45   ±  6.84 | 7   (5, 10) | 57.21   ±  60.93 | 32.85   (8.35, 87.08) | 54 | 11.00% |
| 46 | Romania | 2nd Department of Internal Medicine, Discipline of Diabetes, Nutrition and Metabolic Diseases “Victor Babes” University of Medicine and Pharmacy, Timisoara | YES | 228 | 6.85   ±  4.15 | 6   (4, 9) | 24.05   ±  NA | 24.05   (24.05, 24.05) | 10 | 4.41% |
| 47 | Romania | Carol Davila University of Medicine and Pharmacy Bucharest, Emergency Hospital of Bucharest | NO | 202 | 10.32   ±  12.37 | 6   (4, 11) | 130.4   ±  146.88 | 66   (30, 185) | 27 | 13.37% |
| 48 | Romania | Central Military Emergency Hospital Dr. Carol Davila | NO | 310 | 9.1   ±  7.11 | 7   (5, 11) | 72.63   ±  81.66 | 32.76   (12.4, 112.57) | 57 | 18.39% |
| 49 | Romania | Clinical Emergency Hospital, Bucharest | NO | 122 | 6.59   ±  5.84 | 5   (4, 7.75) | 71.78   ±  69.74 | 42.5   (18.5, 115) | 2 | 1.64% |
| 50 | Romania | Clinical Institute Fundeni, Bucharest | NO | 130 | 13.75   ±  9.57 | 10   (8, 16) | 43.46   ±  48.58 | 28.6   (14, 48.8) | 70 | 53.85% |
| 51 | Romania | Gastroenterology Department, Colentina Clinical Hospital | NO | 126 | 7.73 ± 5.04 | 6   (5, 9) | 45.83   ±  55.58 | 23   (12.12, 55) | 6 | 4.76% |
| 52 | Romania | Maros Megyei Sürgősségi Kórház, Targu Mures | NO | 52 | 8.38   ±  4.08 | 7   (6, 10) | NaN   ±  NA | NA   (NA, NA) | 0 | 0% |
| 53 | Romania | Faculty of Medicine, Ovidius University of Constanta, Romania | NO | 221 | 7.79 ± 4.80 | 7 (5, 10) | 8.06 ± 10.04 | 4.15 (1.64, 10.75) | 15 | 6.78 |
| 54 | Russia | Department of Surgical Disciplines, Immanuel Kant Baltic Federal University, Regional Clinical Hospital, Kaliningrad | NO | 94 | 8.64   ±  4.64 | 8   (6, 11) | 59.75   ±  11.84 | 56   (54.5, 61.25) | 8 | 8.51% |
| 55 | Russia | Sechenov University, Moscow | NO | 17 | 20.53   ±  13.86 | 21   (10, 25) | 4.17   ±  4.37 | 3   (1.75, 6) | 4 | 23.53% |
| 56 | Russia | Omsk State Medical University | NO | 1572 | 10.52   ±  2.62 | 11   (9, 12) | 36.6   ±  17.4 | 34   (24, 47) | 110 | 7.00% |
| 57 | Russia | Sklifosovsky Institute for Clinical Medicine, Sechenov University, Moscow | NO | 20 | 12.35   ±  8.05 | 12   (8, 13) | 89.3   ±  60.72 | 66.85   (61.15, 95) | 4 | 20.00% |
| 58 | Russia | Saint Luke Clinical Hospital, St. Petersburg | NO | 28 | 14.07   ±  15.1 | 11   (8.75, 15) | NaN   ±  NA | NA   (NA, NA) | NA | NA |
| 59 | Serbia | Department for Hepatobiliary and Pancreatic Surgery, Clinic for General Surgery, Military Medical Academy, University of Defense, Belgrade | YES | 99 | 13.9   ±  11.03 | 10   (7, 17) | 56.37   ±  85.93 | 22.88   (13.98, 45.88) | 10 | 10.10% |
| 60 | Slovakia | 1st Department of Surgery UNLP, Kosice | YES | 411 | 11.31   ±  10.93 | 9   (6.5, 13) | 50.37   ±  59.13 | 28.56   (10.62, 72.92) | 29 | 7.06% |
| 61 | Slovakia | Hepatology and Gastroenterology departement of Roosvelt Hospital, Banska Bystrica | NO | 25 | 8.52   ±  6.72 | 6   (4, 10) | 84.35   ±  90.4 | 57.63   (21.19, 117.21) | 2 | 8.00% |
| 62 | Slovakia | University hospital in Martin, Jessenius medical faculty Commenius University, Bratislava | NO | 74 | 10.05   ±  7.22 | 8   (6, 12) | 31.24   ±  41.9 | 13.15   (6.55, 40.15) | 9 | 12.16% |
| 63 | South Korea | Department of Internal Medicine, Jeju National University College of Medicine, Jeju | YES | 80 | 6.06   ±  2.4 | 6   (4, 7) | 64.29   ±  68.02 | 58.4   (12.65, 77.5) | 2 | 2.50% |
| 64 | South Korea | Gachon University, Incheon | NO | 441 | 8.45   ±  10.64 | 6   (4, 9) | 17.3   ±  28.39 | 5.81   (1.54, 19.66) | 59 | 13.38% |
| 65 | Spain | Gastroenterology Department, University Hospital of Santiago de Compostela | NO | 258 | 6.71   ±  7.82 | 5   (3, 7) | 61.79   ±  66.74 | 36.8   (13.02, 95.16) | 16 | 6.20% |
| 66 | Spain | Hospital Vall d'hebron, Barcelona | NO | 454 | 16.35   ±  22.03 | 10   (6, 17) | 6.73   ±  8.56 | 3.15   (0.98, 9.28) | 57 | 12.56% |
| 67 | Spain | Sant Pere de Ribes, General Surgery, Consorci Sanitari del Garrof | NO | 27 | 6.22   ±  3.14 | 5   (4.5, 7.5) | 66.78   ±  87.39 | 16   (1.8, 158.3) | NA | NA |
| 68 | Spain | Servicio de Aparato Digestivo Hospital Clínico Universitario Valladolid | NO | 438 | 9.37   ±  13.91 | 6   (4, 9) | 70.55   ±  75.36 | 43.4   (15.25, 104) | 24 | 5.48% |
| 69 | Spain | University Hospital Arnau de Vilanova - University Hospital Santa María | NO | 267 | 7.8   ±  6.71 | 5   (4, 9) | 82.15   ±  82.09 | 51.8   (21.2, 115.9) | 35 | 13.11% |
| 70 | Tunisia | Habib Bourguiba University Hospital, Sfax | NO | 59 | 12.37   ±  7.59 | 12   (7, 15.5) | 32.01   ±  51.9 | 11.5   (4.6, 18.4) | 2 | 3.39% |
| 71 | Tunisia | Mohamed Ben Sassi Hospital, Gabes | NO | 41 | 9.95   ±  8.97 | 8   (6, 10) | 19.9   ±  44.12 | 8   (5, 15) | 5 | 12.20% |
| 72 | Turkey | Department of Gastroenterology, Sakarya University Faculty of Medicine, Sakarya | NO | 275 | 4.83   ±  3.68 | 4   (3, 6) | 66.29   ±  61.78 | 50   (17.7, 100) | 36 | 13.09% |
| 73 | Turkey | Hospital of Bezmialem Vakif University, School of Medicine, Istanbul | NO | 20 | 3.85   ±  1.18 | 3   (3, 5) | 37.9   ±  62.62 | 19.57   (11.57, 27.91) | NA | NA |
| 74 | Turkey | İzmir Katip Çelebi University Atatürk Training and Research Hospital | NO | 200 | 10.87   ±  10.88 | 8   (5, 14) | 56.35   ±  54.57 | 41.7   (16.85, 85.7) | 39 | 19.50% |
| 75 | Turkey | Marmara University Education and Traning Hospital, Istanbul | YES | 243 | 6.88   ±  7.83 | 5   (4, 7) | 44.16   ±  47.63 | 22.6   (8.59, 69.6) | 46 | 18.93% |
| 76 | Turkey | Dep. of Gastroenterology, Mersin University | YES | 202 | 5.48   ±  3.88 | 4   (3, 6.75) | 63.35   ±  67.39 | 32   (9, 104) | 16 | 7.92% |
| 77 | Ukraine | 1st Department of General Surgery, Bogomolets National Medical University, Kyiv | NO | 198 | 36.9   ±  22.06 | 35   (18, 51) | 36.98   ±  55.48 | 23.15   (16.5, 32.2) | 30 | 15.15% |
| 78 | Ukraine | Dep. of Surgery with a Course of Emergency and Vascular Surgery at Bogomolets National Medical University, Kyiv | NO | 329 | 15.02   ±  18.17 | 10   (8, 15) | 59.36   ±  12.33 | 59   (53, 67) | 9 | 2.74% |
| 79 | Ukraine | Lviv Regional Clinical Hospital | NO | 133 | 15.68   ±  11.74 | 14   (9, 17) | 62.91   ±  98.18 | 35.2   (17.35, 67.25) | 6 | 4.51% |
| 80 | United Kingdom | Liverpool University Hospitals NHS Foundation Trust | NO | 733 | 12.54   ±  21.19 | 7   (4, 12.25) | 47.46   ±  59.53 | 25   (8, 62) | 33 | 4.50% |

List of the centres and their relevant clinical parameters which participated either in the international survey or national cohort analysis. Length of hospitalization is expressed in days, while the discharge CRP value in mg/l.

# TABLE S3. Table of reported discharge protocols

This table describes the main elements of the reported discharge protocols in the international survey. While the abdominal status was mentioned in all case, the assessment of appetite or fever were hardly mentioned-.

#
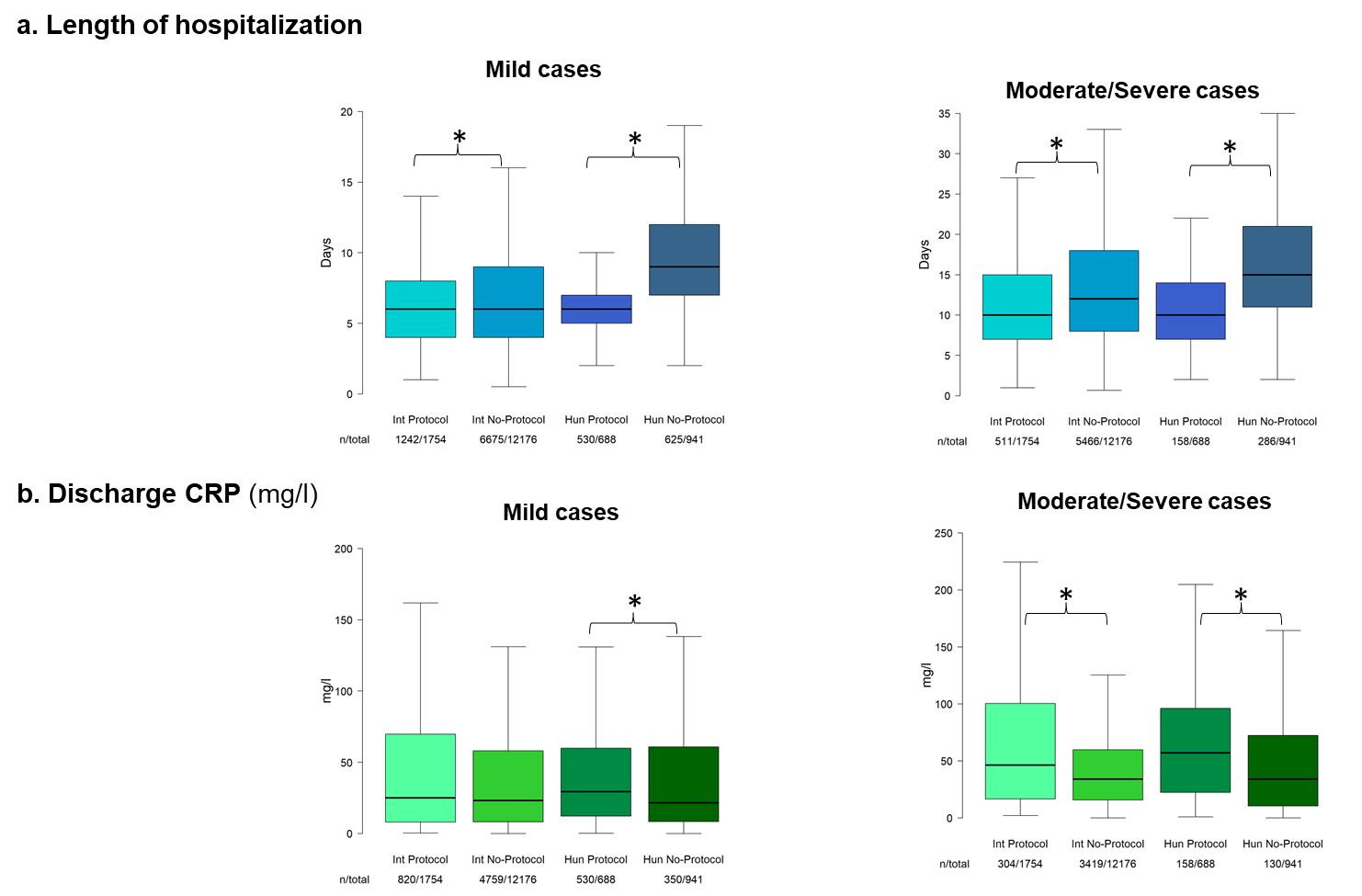
FIGURE S2. Length of hospitalisation and discharge CRP values based on severity visualized by boxplots

There is a significantly shorter LOH and higher discharge CRP in centres with protocolized discharge approach. *p<0.05

# FIGURE S3. Line chart showing the change of CRP level after discharge until 1-month visit


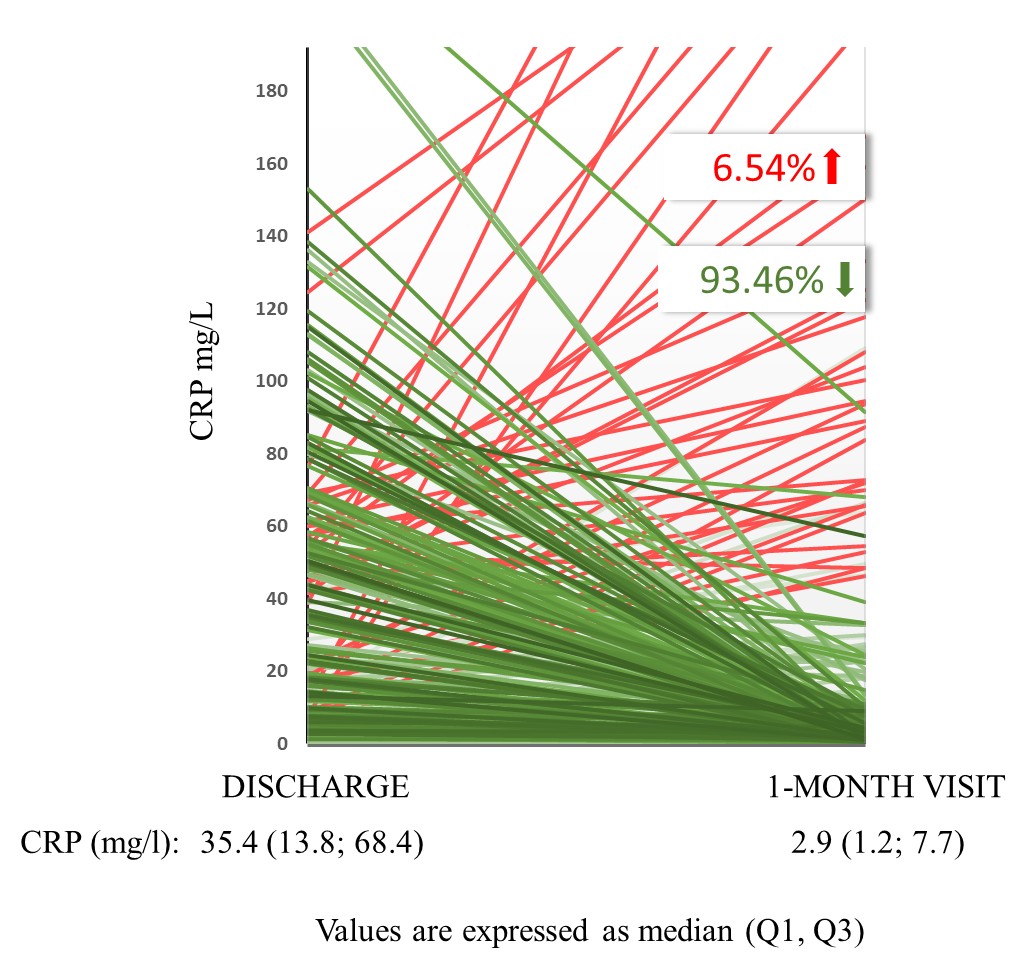


93.46% of the patients presented with a clearly decreasing CRP level at the 1-month follow-up visit compared. Those with elevated CRP level were investigated further.

# TABLE S4. Data quality

International cohort Hungarian cohort

|  | **N** | **Reported data** | **%** |
| --- | --- | --- | --- |
| **Age** | 688 | 688 | 100 |
| **Gender** | 688 | 688 | 100 |
| **Severity** | 688 | 688 | 100 |
| **Length of hospitalization** | 688 | 688 | 100 |
| **Etiology** | 688 | 688 | 100 |
| **Maximum CRP** | 688 | 688 | 100 |
| **Discharge CRP** | 688 | 688 | 100 |
| **1-month CRP** | 688 | 688 | 100 |
| **Readmission** | 688 | 688 | 100 |
| **Etiology of readmission** | 35 | 35 | 100 |
| **Total** | **6227** | **6227** | 100 |

|  | **N** | **Reported data** | **%** |
| --- | --- | --- | --- |
| **Age** | 14650 | 14644 | 99 |
| **Gender** | 14650 | 14649 | 99 |
| **Severity** | 14650 | 14628 | 99 |
| **Length of hospitalization** | 14650 | 14599 | 99 |
| **Etiology** | 14650 | 14636 | 99 |
| **In-hospital mortality** | 14650 | 14483 | 98 |
| **Discharge CRP** | 14650 | 9598 | 65 |
| **Readmission** | 14650 | 13948 | 95 |
| **Etiology of readmission** | 1379 | 1195 | 94 |
| **Total** | **119459** | **113370** | **94** |

# FIGURE S4. ROC curve and AUC value representing the sensitivity and specificity of discharge CRP level in terms of readmission in all (a.) and only in mild AP cases (b.).


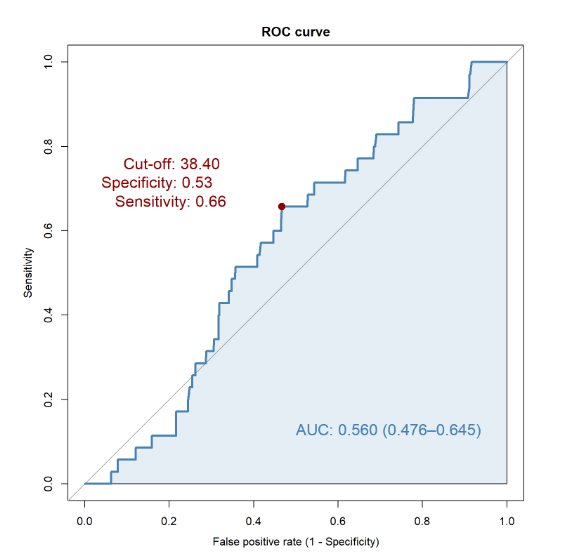

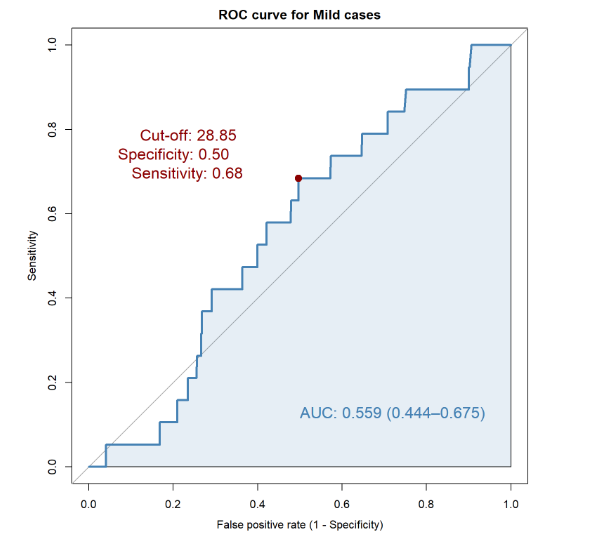


a.

b.

#

The AUC value of 0.560 and 0.559 in the total and only in the mild cases represent a close-to-random state, thus we cannot say with certainty that discharge CRP can predict the readmissions.

# FIGURE S5. Relationship of 24 and 48 hours decreasing tendency in CRP and readmisson rates


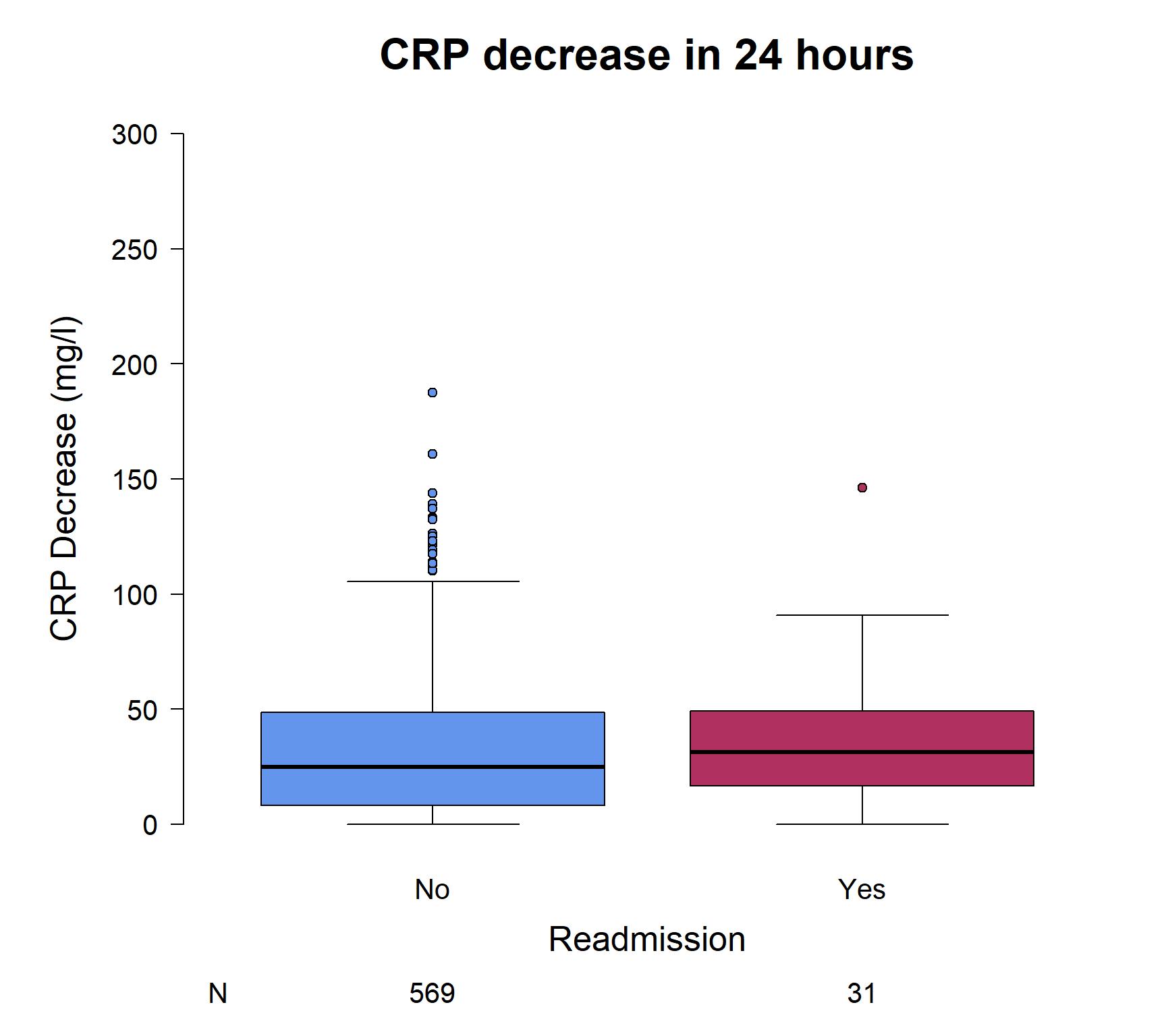

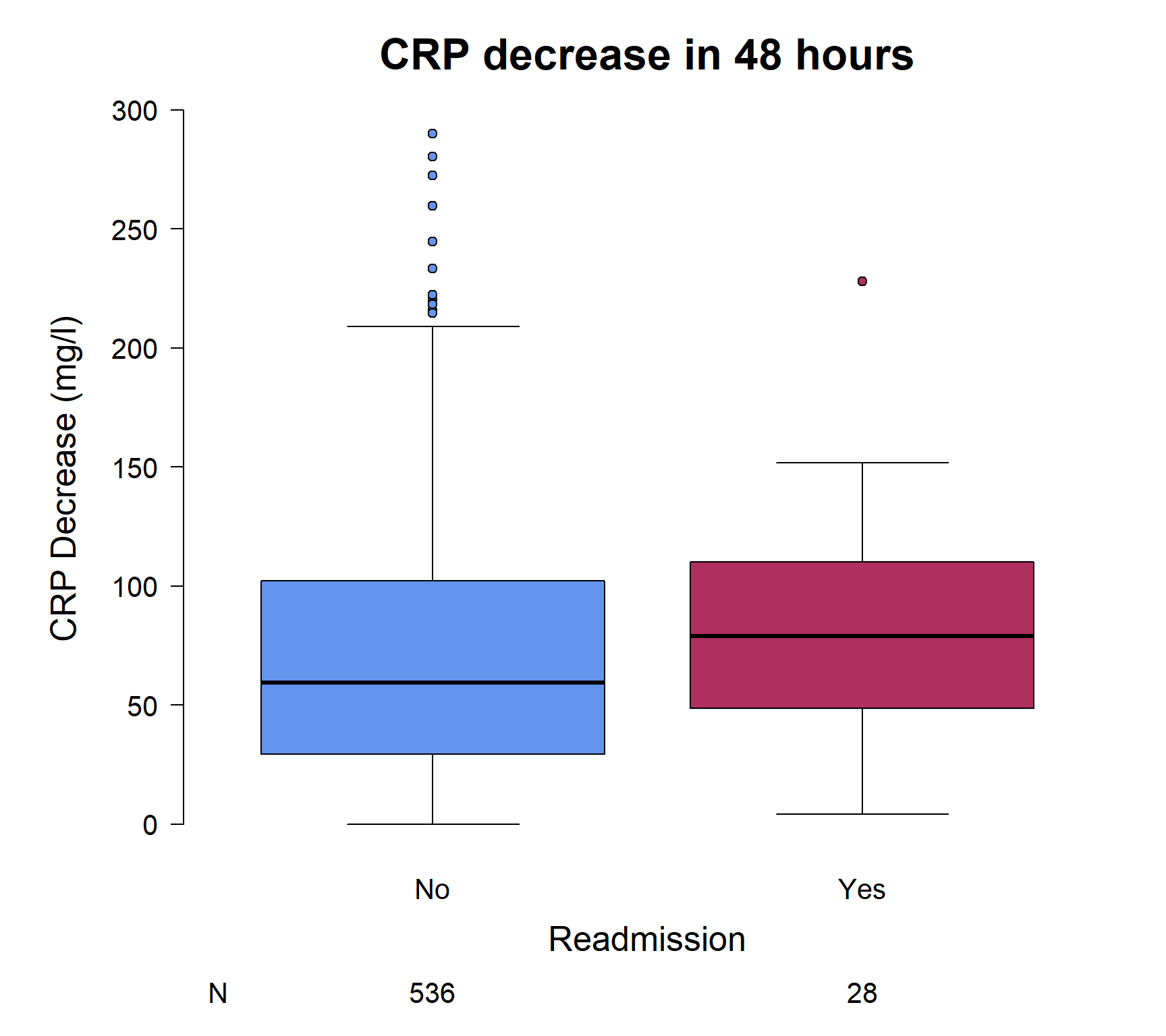


p=0.461

p=0.175

There is no association between the volume of CRP value decrease and the risk of 1-month readmssion.
